# GD2 ganglioside-binding antibody 14G2a and specific aurora A kinase inhibitor MK-5108 induce autophagy in IMR-32 neuroblastoma cells

**DOI:** 10.1007/s10495-018-1472-9

**Published:** 2018-07-19

**Authors:** Małgorzata Durbas, Paweł Pabisz, Katarzyna Wawak, Aneta Wiśniewska, Elżbieta Boratyn, Iwona Nowak, Irena Horwacik, Olga Woźnicka, Hanna Rokita

**Affiliations:** 1 Laboratory of Molecular Genetics and Virology, Faculty of Biochemistry, Biophysics and Biotechnology, Jagiellonian University, Gronostajowa 7, 30-387 Kraków, Poland; 2 Departament of Biophysics, Faculty of Biochemistry, Biophysics and Biotechnology, Jagiellonian University, Gronostajowa 7, 30-387 Kraków, Poland; 3 Department of Cell Biology and Imaging, Faculty of Biology, Institute of Zoology, Jagiellonian University, Gronostajowa 9, 30-387 Kraków, Poland

**Keywords:** Neuroblastoma, Ganglioside GD2, 14G2a monoclonal antibody, Aurora A, Autophagy, Apoptosis

## Abstract

The process of autophagy and its role in survival of human neuroblastoma cell cultures was studied upon addition of an anti-GD2 ganglioside (GD2) 14G2a mouse monoclonal antibody (14G2a mAb) and an aurora A kinase specific inhibitor, MK-5108. It was recently shown that combination of these agents significantly potentiates cytotoxicity against IMR-32 and CHP-134 neuroblastoma cells in vitro, as compared to the inhibitor used alone. In this study we gained mechanistic insights on autophagy in the observed cytotoxic effects exerted by both agents using cytotoxicity assays, RT-qPCR, immunoblotting, and autophagy detection methods. Enhancement of the autophagy process in the 14G2a mAb- and MK-5108-treated IMR-32 cells was documented by assessing autophagic flux. Application of a lysosomotropic agent—chloroquine (CQ) affected the 14G2a mAb- and MK-5108-stimulated autophagic flux. It is our conclusion that the 14G2a mAb (40 μg/ml) and MK-5108 inhibitor (0.1 μM) induce autophagy in IMR-32 cells. Moreover, the combinatorial treatment of IMR-32 cells with the 14G2a mAb and CQ significantly potentiates cytotoxic effect, as compared to CQ used alone. Most importantly, we showed that interfering with autophagy at its early and late step augments the 14G2a mAb-induced apoptosis, therefore we can conclude that inhibition of autophagy is the primary mechanism of the CQ-mediated sensitization to the 14G2a mAb-induced apoptosis. Although, there was no virtual stimulation of autophagy in the 14G2a mAb-treated CHP-134 neuroblastoma cells, we were able to show that PHLDA1 protein positively regulates autophagy and this process exists in a mutually exclusive manner with apoptosis in *PHLDA1*-silenced CHP-134 cells.

**Electronic supplementary material:**

The online version of this article (10.1007/s10495-018-1472-9) contains supplementary material, which is available to authorized users.

## Introduction

Neuroblastoma is the most common extracranial cancer in children [1]. High-risk neuroblastoma, documented to occur in half of all patients, is typically associated with metastasis to the liver, bone or bone marrow, and aggressive features such as *MYCN* oncogene amplification, as well as an unfavorable prognosis [2]. Treatment of children > 18 months of age with widely disseminated neuroblastoma (stage 4) and those < 18 months with *MYCN*-amplified stage 4 disease remains one of the greatest challenges for pediatric oncologists [3]. The treatment methods used in the management of high-risk neuroblastoma include chemotherapy, surgery, myeloablative therapy and stem cell transplant, radiotherapy, and use of agents such as ch14.18 in combination with IL-2/GM-CSF and isotretinoin [3, 4]. Ch14.18 monoclonal antibodies, being IgG1 human/mouse chimeric switch variant of murine 14G2a monoclonal antibody bind to GD2 ganglioside. GD2 is one of the most important molecular targets for immunotherapy in neuroblastoma. This is a surface glycolipid, highly expressed on neuroblastoma cells with only limited distribution on healthy tissues. Studies have shown that mAbs targeting GD2 inhibit tumor cell growth not only by means of immunological mechanisms such as antibody-dependent cell-mediated cytotoxicity and complement-dependent cytotoxicity, but also directly through cell death induction [5]. These features stress the potential for finding effective GD2-targeted therapies using monoclonal antibodies and their combinations with other agents for use in high-risk neuroblastoma patients. We have previously shown that the anti-GD2 14G2a mouse monoclonal antibody decreases survival of IMR-32 human neuroblastoma cells in a dose-dependent manner. Cell death evoked by this antibody exhibited several characteristics typical for apoptosis with partially caspase-dependent mechanism [6].

Another important target in neuroblastoma, which has gained wide popularity, is aurora A kinase. It is required for centrosome maturation and division, entry into mitosis and formation of the mitotic spindle [7]. The gene encoding aurora A kinase is amplified in multiple human tumors such as breast, cervical or gastric tumors and neuroblastoma [7]. Overexpression of the *AURKA* gene in neuroblastoma is associated with high-risk, late-stage tumors, unfavorable histology, *MYCN* amplification, and in general decreased survival of neuroblastoma patients [8]. Therefore, aurora A kinase has been extensively studied as an antimitotic drug target. Several specific inhibitors have been developed and are currently evaluated in preclinical models as well as in different phases of clinical trials [9]. The effects of aurora A kinase inhibition are multiple, and include events such as abnormal spindle pole formation, cell-cycle arrest between phases G2-M and polyploidy, followed by induction of apoptosis [7]. Aurora A forms a complex with MYCN in *MYCN*-amplified neuroblastoma cells, protecting MYCN from proteasomal degradation during mitosis [10]. Therefore, inhibition of aurora A kinase may be an effective strategy to treat *MYCN*-amplified neuroblastoma. Our previous study showed that a small molecule, specific aurora A kinase inhibitor, MK-5108, decreases neuroblastoma cell survival, and when used in combination with the 14G2a mAb, significantly potentiates cytotoxicity against neuroblastoma cells in vitro, as compared to MK-5108 used alone [11].

Recently evidence mounts on how another essential cellular process, autophagy, may be involved in the tumor biology. Autophagy is a homeostatic, catabolic degradation process responsible for cellular proteins and organelles being engulfed by autophagosomes, digested in lysosomes, and recycled to sustain cellular metabolism [12]. Autophagy was shown to have dual roles in cancer, acting both as a tumor suppressor by preventing the accumulation of damaged proteins and organelles and as a mechanism of cell survival that can promote the growth of established tumors [13, 14]. Tumor cells induce autophagy in response to cellular stress and/or increased metabolic demands resulting from rapid cell proliferation. Autophagy-associated tolerance to stress enables cell survival by maintaining energy production that can lead to tumor growth and drug resistance [15]. As shown in preclinical models, inhibition of autophagy restored chemosensitivity and enhanced tumor cell death [16]. These results established autophagy as a therapeutic target and led to multiple clinical trials in humans to evaluate the effect of autophagy inhibition using hydroxychloroquine in combination with chemotherapy or targeted agents [17]. Contrary to the cytoprotective function of autophagy, which is supported by abundant evidence, prolonged stress and sustained autophagy may eventually lead to cell death when protein and organelle turnover overwhelm the capacity of the cell. In cancer cells, autophagy accompanied by nonapoptotic cell death has also been described [18, 19]. As autophagy is such a fundamental process, it is very important to establish how treatment strategy for neuroblastoma influences the functional status of autophagy. This is especially critical, as many current cancer therapeutics activate autophagy. Therefore, efforts to understand and modulate the autophagy pathway could provide new approaches to cancer therapy and prevention.

This study aimed at further elucidation of mechanisms governing cytotoxic activity of the anti-GD2 monoclonal antibody and the aurora A kinase specific inhibitor in the IMR-32 and CHP-134 human neuroblastoma cell lines, especially in the context of the possible stimulation of autophagy. Firstly, we showed that both agents induce autophagy in IMR-32 cells. Secondly, we studied the role of autophagy process in the 14G2a mAb-induced cell killing of IMR-32 cells. We thoroughly characterized the crosstalk between intracellular processes such as autophagy and apoptosis modulated by the 14G2a mAb in IMR-32 cells. Finally, we explored a role of PHLDA1 protein in regulation of autophagy in CHP-134 neuroblastoma cells. We confirmed that PHLDA1 is able to positively regulate autophagy in the cell line.

## Materials and methods

### Cell culture

IMR-32 human neuroblastoma cell line was cultured in EMEM medium (M4655, Sigma-Aldrich) supplemented with 10% fetal calf serum (10270106, Gibco), 1% non-essential amino acid solution (M7145, Sigma-Aldrich), 1 mM sodium pyruvate (S8636, Sigma-Aldrich) and 50 µg/ml gentamicin (G1397, Sigma-Aldrich). CHP-134 cells were grown in RPMI 1640 medium (R8758, Sigma-Aldrich) supplemented with 10% FCS and 50 µg/ml gentamicin. LAN-1 cells were cultured in EMEM/F-12 (N6658, Sigma-Aldrich) medium diluted in 1:1 ratio, supplemented with 10% FCS, 1% non-essential amino acid solution, 1 mM sodium pyruvate and 50 µg/ml gentamicin, while LAN-5 cells in RPMI 1640 medium supplemented with 20% FCS and 50 µg/ml gentamicin. For preparation of positive controls, IMR-32 and CHP-134 cells were cultured in amino acids-deprived Earle’s Balanced Salt (E2888, Sigma-Aldrich), supplemented with 10% FCS for 24 h. All cell lines were grown at 37 °C in a 5% CO_2_ atmosphere.

### Antibody purification

Mouse GD2-binding mAbs, 14G2a (IgG2a) were purified from 14G2a hybridoma culture supernatants using the HiTrap Protein G HP column (17-0404-01, GE Healthcare Bio-Sciences AB) according to the manufacturer’s protocol. Antibodies were dialysed using D-Tube ^TM^ Dialyzer Midi tubes (71507-3, Millipore) against 4 liters of PBS (phosphate-buffered saline, pH 7.3–7.5) for 24 h at 4 °C. Protein concentration was measured using the BCA assay (B9643, C2284, Sigma-Aldrich) according to the manufacturer’s protocol.

### Drugs treatment

IMR-32 and CHP-134 cells were pretreated with indicated concentration of chloroquine diphosphate salt (further abbreviated to CQ, C6628, Sigma-Aldrich) or bafilomycin A1 (further abbreviated to Baf, B1793, Sigma-Aldrich) for 1.5 h at 37 °C and subsequently treated for a given time with either the 14G2a mAb at concentration of 40 μg/ml, or MK-5108 (further abbreviated to MK, S2770, Selleckchem) at concentration of 0.1 μM, seeded, and grown at 37 °C. Additionally, PBS-treated, water-treated or DMSO-treated control cells were included. Control cells were treated with equivalent volume of the solvent of the drug.

### Cell viability tests

2 × 10^5^ of IMR-32 cells/well and 5 × 10^3^ of CHP-134 cells/well were cultured on 96-well plates, and treated with given agents for 24, 48 or 72 h (with prior incubation with autophagy inhibitors). Cellular ATP content was measured using ATPlite Luminescence ATP Detection Assay System (6016947, PerkinElmer) according to manufacturer’s protocol using the Infinite M200 Reader (Tecan Group Ltd.).

### Measurements of caspase 3/7 activity

For measurements of caspase 3/7 activity, 2 × 10^5^ of IMR-32 cells/well were cultured for 3 days in 100 μl of complete medium in a 96-well plate, after a given treatment. On the third day, cell cultures were analyzed using Caspase-Glo® 3/7 Assay (G8090, Promega) according to the manufacturer’s protocol. Samples luminescent signals were analyzed in triplicate using the Infinite M200 Reader.

### RNA isolation and RT-qPCR

For RNA isolation, 1 × 10^6^ of IMR-32 cells/well were grown in 5 ml of culture medium and 2.5 × 10^5^ of CHP-134 cells/well were grown in 5 ml of culture medium in 6-well plates. Total RNA was extracted using TRI-REAGENT® (TRI118, Lab Empire), as described in manufacturer’s protocol (Molecular Research Center, Inc.). 2 µg of total RNA was reverse-transcribed. Following the synthesis, cDNA was amplified in qPCR using the Eco Illumina (Illumina) system. For the normalization of each sample, ribosomal protein S13 (RPS13) cDNA was used. Quantification was performed using the “ΔΔCt” relative quantitation method. All samples were run in triplicate. Primers used for qPCR reaction were designed using Primer-BLAST (http://www.ncbi.nlm.nih.gov/tools/primer-blast/), unless otherwise stated, and were as follows: *LC3B* (F: GATGTCCGACTTATTCGAGAGC, R: TTGAGCTGTAAGCGCCTTCTA); *ATG7* (F: ACCCAGAAGAAGCTGAACGA, R: CTCATTTGCTGCTTGTTCCA); *BCN1* (F: AGGATGATGTCCACAGAAAGTGC, R: AGTGACCTTCAGTCTTCGGCTG); *ATG12* (F: GCGAACACGAACCATCCAAG, R: CCATCACTGCCAAAACACTCAT); *ATG5* (F: GGTGAAGGTGGTTCCTCCG, R: AGCCAAACTTAGTAAGCAACAGAC), *ATG16L* (F: GCATGACGTACCAAACAGGC, R: ATCACCAGTTGAGCTCCCCA), *RPS13* (F: TCGGCTTTACCCTATCGACGCAG, R: ACGTACTTGTGCAACACCATGTGA) [20] and *PHLDA1* (F: TGCCTGAAAGGGGCAGCTCC, R: TGATCTGGTGCGGGGCGGA) as described in [21].

### Protein isolation and immunoblotting

For protein analysis, the IMR-32 (1 × 10^6^) and CHP-134 (0.25 × 10^6^) cells/well were grown on 6-well plates. Whole cell extracts were obtained according to the TRI-REAGENT® manufacturer’s protocol. The protein lysates were resolved in the denaturing SDS-PAGE, and transferred onto a Immobilon®-P Transfer Membrane (IPVH00010, Millipore). The membranes were treated with a blocking solution containing 10 mM Tris (pH 7.4), 150 mM NaCl, 0.05% Tween 20 and 5% nonfat dry milk for 1 h at room temperature, and incubated overnight with the respective primary antibody at 4 °C. After the washing steps, the membranes were treated with the appropriate secondary antibody for 1 h at room temperature. The immunoreactive bands were visualized using a chemiluminescence method (WBKLSO100, Immobilon Western HRP Substrate, Millipore) according to the manufacturer’s protocol. Chemiluminescence was detected with MicroChemi system (DNR Bio-Imaging). The intensity of the immunoreactive bands was determined by densitometric scanning to quantify changes in the protein levels and analyzed by Quantity One Analysis Software (BioRad). The values for analyzed protein among samples were normalized using the respective values for α-tubulin. The level of the protein expression in control samples was set as 1. The following monoclonal antibodies against: Beclin-1 (#3495); LC3A/B (#12741); ATG5 (#12994); ATG12 (#4180); ATG16L1 (#8089); ATG7 (#8558); ATG3 (#3415); P62/SQSTM1 (#5114); α-tubulin (#2125); cleaved caspase-3 (#9664); cleaved PARP (#5625); PARP (#9542); Bcl-xl (#2764); Bax (#5023) and IgG, HRP-linked antibody (#7074) were purchased from Cell Signaling.

### Fluorescent detection of autophagic compartments

IMR-32 (1 × 10^6^) and CHP-134 (2.5 × 10^5^) cells/well were pretreated with CQ and subsequently treated with respective drugs (14G2a mAb, MK-5108 or their respective controls), seeded at 6-well plate and after given time prepared for detection of autophagic compartments with the CYTO-ID dye according to the manufacturer’s protocol (ENZ-51031, CYTO-ID® Autophagy Detection Kit, ENZO). Briefly, cells were collected to 1.5 ml tubes after the treatment and washed with 1× Assay Buffer. Next, cells were stained with the CYTO-ID dye for detection of autophagic compartments and counterstained with Hoechst 33342 Nuclear Stain for 30 min at 37 °C in the dark. After staining, cells were washed two times and used for measurements using a fluorescence microplate reader (1:1000 dilution of each dye in 1× Assay Buffer) and for florescence microscopy application (1:2000 dilution of CYTO-ID® and 1:1000 dilution of Hoechst 33342 in 1× Assay Buffer). For microplate reader measurements, cells were re-suspended in 1× Assay Buffer, seeded on a 96-well black plate in triplicate (2.5 × 10^5^ cells per 100 μl) and the signals were analyzed with a fluorescence microplate reader (CLARIOstar®, BMG LABTECH). Relative mean fluorescence intensity of CYTO-ID was monitored at 24, 48 and 72 h and divided by relative ATP level of the respective groups of cells (to normalize signals to number of viable cells). For fluorescence microscopy application, cells were fixed in 4% paraformaldehyde for 20 min, washed three times and drops of cell suspensions were applied onto glass microscope slides, overlaid with coverslips, and visualized under the fluorescent microscope (Carl Zeiss Microscopy, LLC). The standard FITC (excitation ~ 480 nm, emission ~ 530 nm) and DAPI (excitation ~ 340 nm, emission ~ 480 nm) filters were set for imaging the autophagic and nuclear signals. Relative mean fluorescence intensity of CYTO-ID-stained autophagic compartments was quantified in five randomly selected photomicrographs (taken using a 40× objective). ImageJ 1.50i software (Wayne Rasband, National Institutes of Health, USA) was used for quantification of the dyes fluorescence.

### Electron microscopy

IMR-32 (1 × 10^6^) and CHP-134 (2.5 × 10^5^) cells/well were pretreated with CQ and subsequently treated with the 14G2a mAb or PBS and seeded at a 6-well plate. After 48 h cells were prepared for detection of autophagic compartments by transmission electron microscopy (TEM). IMR-32 and CHP-134 cells were fixed with 2.5% glutaraldehyde in 0.1 M cacodylic buffer, washed with 0.1 M cacodylic buffer, followed by 1% OsO_4_ (1 h). After dehydration in graded ethanol (50% 1 × 10 min, 70% 1 × 10 min, 96% 1 × 10 min, 100% 2×/15 min) and propylene oxide 2 × 10 min, samples were gradually infiltrated with resin Poly/Bed812 mixed with propylene oxide—2:1 (2 h) and 1:1 (overnight). Next, samples in fresh resin were put to oven for polymerization at 60 °C for 3 days. The samples were cut using Microtome (Leica). The thin sections (approximately 70 nm) on one slot with formvar covered by carbon were stained with uranyl acetate and lead citrate for observation using JEOL 2100HT TEM (JEOL). The percentage of residual bodies scattered along cytoplasm on a cell cross-section was measured by placing a net (20 pxl × 20 pxl) on the image (2048 pxl × 2048 pxl). The net was generated in Photo Filtre 7 software, where the number of squares per the area of residual bodies (x) and the cell (y) was counted. The percentage of residual bodies was calculated as ∑x/∑y × 100%. The mean from ten images was taken for calculations.

### siRNA transfection

1.5 × 10^6^ of IMR-32 cells/well were seeded in 2.5 ml of complete medium on 6-well plates, 24 h prior to siRNA transfection. On the day of transfection, *Silencer*® Select siRNAs sequences (Ambion) either for *ATG7* silencing (#4392420) or control (Mock) siRNAs (#4390843) were added to Opti-MEM medium (319850-047, Gibco). A Lipofectamine® RNAiMAX Reagent (#13778030, Ambion) was used according to the manufacturer’s recommendations, with diluted Lipofectamine® RNAiMAX Reagent: diluted siRNAs ratio of 1:1 in Opti-MEM medium. 250 μl of the transfection mix was added to 2.5 ml of complete fresh medium at the final siRNAs concentration of 10 nM. One well contained cells that were not transfected (WT). For the purpose of study relying on early interference with autophagy, 24 h after transfection with *ATG7*-targeting siRNAs or control siRNAs, cells were treated with combination of the 14G2a mAb and/or CQ for the next 24 h. 24 and 48 h after transfection (depending on the described type of treatment) ATP and caspase 3/7 activity was measured and cells were collected for subsequent RNA and protein isolation.

### shRNA leniviral particles transduction

CHP-134 cells (5 × 10^4^) were plated in 2 ml of complete medium in a 12-well plate, 24 h prior to viral infection. On the day of infection media were replaced with 2 ml of complete medium with Polybrene® (sc-134220, Santa Cruz Biotechnology) at the final concentration of 5 µg/ml. For the *PHLDA1* gene silencing, cells were infected by adding 40 µl of the shRNA Lentiviral Particles stock (sc-36631-V, Santa Cruz Biotechnology) to the culture. Additionally, one well with cells was transduced with Control shRNA Lentiviral Particles (sc-108080, Santa Cruz Biotechnology) and one well with cells was transduced with copGFP Control Lentiviral Particles (sc-108084, Santa Cruz Biotechnology) for measuring efficiency of transduction. Finally, one well contained cells that were not transduced (WT). On the third day media were replaced with 2 ml of fresh complete medium without Polybrene. On the fourth day, puromycin dihydrochloride (sc-108071, Santa Cruz Biotechnology) was added to *PHLDA1*-silenced and control (Mock) cells to the fresh media at the concentration of 5 µg/ml. To derive *PHLDA1*-stably silenced clones, media were replaced with fresh medium containing puromycin every 3–4 days, until resistant colonies were selected. Then, these colonies were transferred with small pipette tips to new culture plates and expanded. The effects of *PHLDA1*-silencing were assessed by RT-qPCR and confirmed by western blot analyses.

### Statistical analyses

Data was presented as means ± SEM. All experiments were repeated at least three times and series of pairwise tests (*t* test) were performed, comparing means of treated cells versus control cells, set as 1 (black baseline). For IC_50_ calculations, curves were fitted using dose response model with the OriginPro9.1 software.

## Results

### Analysis of autophagy-related genes and proteins in the 14G2a mAb-treated IMR-32 and CHP-134 cells

**Fig. 1:**
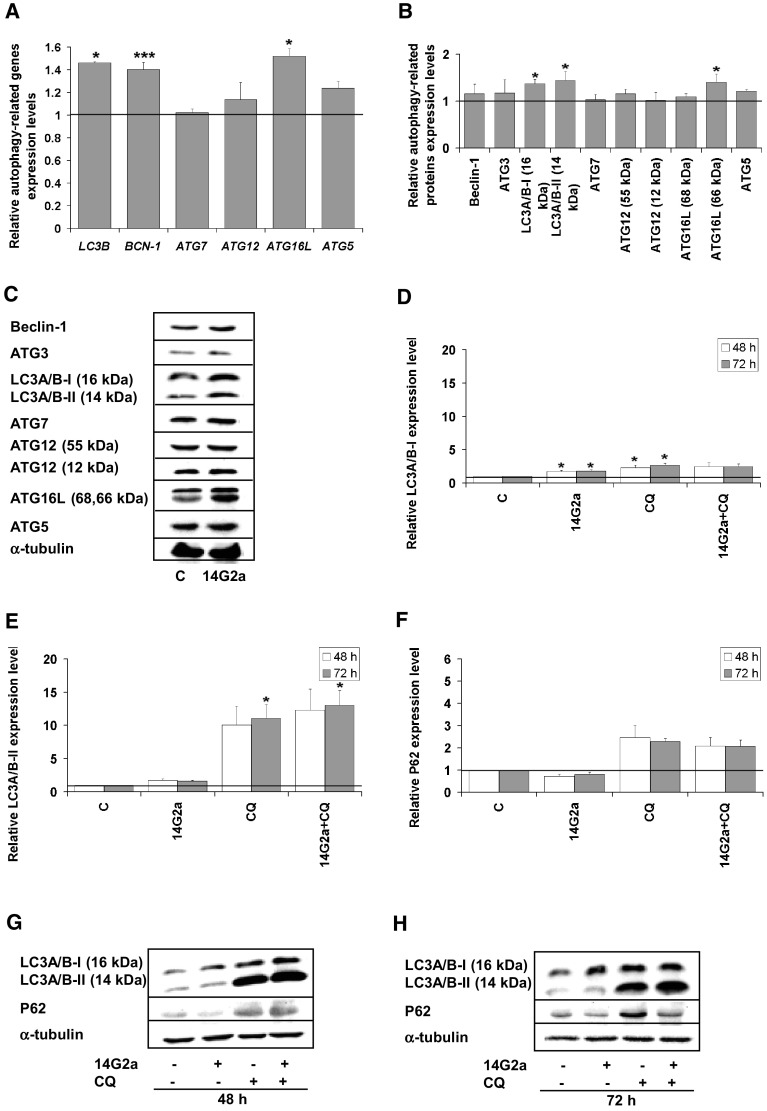
Analysis of autophagy in the 14G2a mAb-treated IMR-32 neuroblastoma cells. **a** Relative gene expression of *LC3B, BCN-1, ATG7, ATG12, ATG16L*, and *ATG5* in the 14G2a mAb-treated IMR-32 cells (in concentration of 40 μg/ml), as compared to the control (PBS-treated cells), assessed at 48 h by RT-qPCR. *RPS13* cDNA was used as the reference. **b** Relative protein expression of Beclin-1, ATG3, LC3A/B-I, LC3A/B-II, ATG7, ATG12, ATG16L, and ATG5 was measured in IMR-32 cells at 48 h by western blot and normalized to α-tubulin. Expression of proteins and their respective genes in the control cells equals 1 (black baseline). **c** Representative immunoblots are presented. (D) Relative LC3A/B-I expression affected by the 14G2a or/and CQ treatment in IMR-32 cells as assessed by western blot. Cells were pre-treated with 10 μM CQ for 1.5 h and subsequently treated with the 14G2a mAb or PBS (control) for 48 and 72 h. **e** LC3A/B-II—estimated autophagy flux affected by the 14G2a mAb or/and CQ treatment in IMR-32 cells as assessed by western blot. **f** Relative P62 expression affected by the 14G2a mAb or/and CQ treatment in IMR-32 cells as assessed by western blot. **g, h** Representative immunoblots are presented. C—control, PBS-treated cells; 14G2a—the 14G2a mAb-treated cells; CQ—chloroquine-treated cells. P-values for *t* test were as follow: *p < 0.05, ***p < 0.001

We assessed expression level of autophagy-associated genes e.g., *LC3B* (encoding for LC3B), *BCN-1* (encoding for Beclin-1) and genes from the *ATG* family (autophagy-related genes) using RT-qPCR (Fig. 1a). We observed the significant increase in the expression of *LC3B* and *BCN-1* genes in the 14G2a mAb-treated IMR-32 cells (at concentration of 40 μg/ml) to approximately 1.4 and non-significant up-regulation of *ATG12* and *ATG5* genes expression, as compared to the control cells. Significant increase in the mRNA expression level of *ATG16L* to 1.5 was also noted, as compared to control, but no change in mRNA expression was visible for *ATG7*. Although, autophagy genes may be transcriptionally upregulated in response to stress conditions that induce autophagy, there is no clear evidence that autophagic activity per se is related to transcriptionally upregulated genes [22]. Moreover, the expression of microtubule-associated protein light chain 3 (MAP-LC3A/B, abbreviated further to LC3A/B), was also measured as a key autophagy marker. LC3A/B is incorporated into the autophagosome membrane and it can be tracked until fusion with the lysosome vesicle [23]. The LC3A/B exists in two forms: the cytoplasmic LC3A/B-I (16 kDa) and the autophagosome-associated, undergoing proteolytic cleavage and modification, LC3A/B-II (14 kDa) form [24]. Indeed, in IMR-32 cells we observed significantly higher level of LC3A/B-II converted form at 48 h, as compared to the control cells as assessed by western blot (Fig. 1b, c). The amount of LC3A/B-II usually correlates well with the number of autophagosomes, therefore the increase of LC3A/B-II expression signal to 1.5 indicates accumulation of autophagosomes in the 14G2a mAb-treated IMR-32 cells. Furthermore, we tracked changes in expression of other proteins related to autophagy pathway i.e., Beclin-1, ATG3, ATG7, ATG12, ATG16L, and ATG5 in the 14G2a mAb-treated and the control IMR-32 cells. We observed a significant up-regulation of ATG16L expression signal to 1.4, slight non-significant increase in ATG5 level and marginal changes in regulation of the other proteins (Fig. 1b, c). However, ATG proteins are constitutively expressed in sufficient amounts, and their posttranslational modifications and/or associations with members of the autophagic machinery, rather than regulation of their expression levels, is critical for their activity in the autophagy pathway [22]. These results allowed us to show that the 14G2a mAb modulates expression of some autophagy-associated molecules in IMR-32 cells on mRNA and protein levels. CHP-134, another neuroblastoma cell line responsive to the 14G2a mAb treatment, was also characterized in terms of regulation of autophagy-associated genes and proteins. However, no significant changes or only a slight decrease in the aforementioned genes mRNA expression and proteins levels were observed in the 14G2 mAb-treated cells, as compared to the control cells (Supplementary Fig. 1a–c). These results suggest no enhancement of autophagy process in the 14G2a mAb-treated CHP-134 cell cultures. Nevertheless, autophagy-related molecules regulated in the 14G2a mAb-treated cells should not be considered as the only indicators for monitoring autophagy and these results are further supported by other types of analyses.

### Monitoring autophagic flux in the IMR-32 and CHP-134 cells

The accumulation of autophagosomes may represent either the increased generation of autophagosomes and/or a block in autophagosomal maturation and the completion of the autophagy pathway [22, 25]. To distinguish between these two scenarios, we performed LC3A/B turnover assay to verify whether autophagosome accumulation is due to autophagy induction or rather a block in downstream steps. The LC3A/B turnover assay is one of the principal methods to measure “autophagic flux” i.e., the amount of LC3A/B that is delivered to lysosomes for degradation [26]. To this end, cells were treated with lysosomotropic reagent—chloroquine diphosphate salt (abbreviated further to CQ), a weak base amine that accumulates in the lysosomal lumen and inhibits the fusion of autophagosomes with lysosomes. Accordingly, the differences in the amount of LC3A/B-II between samples in the presence and absence of CQ represent changes in autophagic flux. If cells are treated with CQ, degradation of LC3A/B-II is partially inhibited, and LC3A/B-II accumulates. The levels of LC3A/B-I (Fig. 1d, g, h) and LC3A/B-II (Fig. 1e, g, h) forms in IMR-32 cells were analyzed by western blot. We showed that autophagic flux occurs in IMR-32 cells treated with the 14G2a mAb because the signal of the level of LC3A/B-II is increased to respectively 1.7 and 1.6 at 48 and 72 h, as compared to the control cells (Fig. 1e, g, h). Treatment with the 14G2a mAb and CQ further increases LC3A/B-II level signals to 12.3 and 13.1, respectively, providing that autophagic flux is affected, manifesting itself by further LC3A/B-II accumulation. This allowed us to confirm an actual induction of autophagy in IMR-32 cells via autophagosomes accumulation, but not via autophagosomes degradation. On contrary, the signal for the LC3A/B-I form increased only to a small extent (to 2.5) for the 14G2a mAb and CQ used (Fig. 1d, g, h), as compared to LC3A/B-II signal, which escalated for the combined treatment. This indicates that pool of the cytoplasmic LC3A/B-I form is intensely converted to the autophagosome-bound LC3A/B-II protein. Expression level of another important autophagy marker, P62 (SQSTM1) protein, was also monitored in time (Fig. 1f–h). Level of this protein is inversely correlated with induction of autophagy, as the P62 protein itself is a subject of degradation in the autolysosome [27]. Therefore, the observed decrease in the P62 signal level to 0.7 in the 14G2a mAb-treated cells, as compared to the control cells and a consecutive decrease of the P62 protein signal to 2.1 in the 14G2a mAb/CQ-treated cells, as compared to CQ-treated cells (2.5), confirm autophagy induction in our model at 48 h (Fig. 1f–h). An overall estimation of autophagy by performing the autophagic flux assay in CHP-134 cells upon the 14G2a mAb treatment showed no elevated autophagic activity in the 14G2a mAb-treated CHP-134 cells, as compared to control (Supplementary Fig. 1d–h).

### Detection of autophagic compartments with the CYTO-ID dye in the IMR-32 and CHP-134 cells

**Fig. 2:**
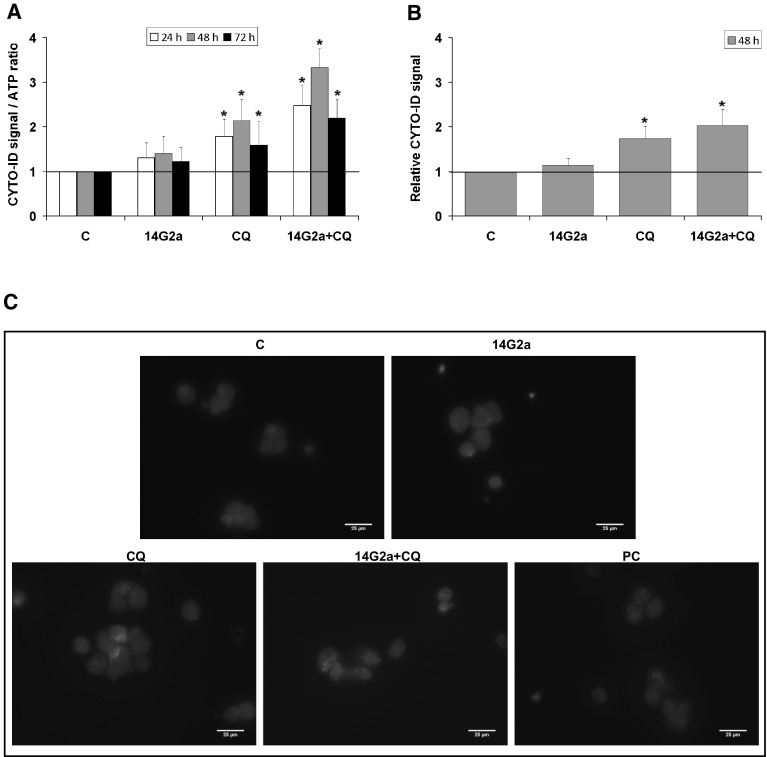
Relevance of autophagy flux in the 14G2a-treated IMR-32 cells. Cells were pre-treated with 10 μM CQ for 1.5 h and subsequently treated with the 14G2a mAb or PBS (control). **a** CYTO-ID—estimated autophagy flux in IMR-32. Relative mean fluorescence intensity of the CYTO-ID dye was measured at 24, 48, and 72 h by a microplate reader and divided by ATP level signals of the respective groups of cells. **b** IMR-32 cells were stained with CYTO-ID and Hoechst 33342, fixed and visualized under the fluorescence microscope. Relative mean fluorescence intensity of CYTO-ID-stained autophagic compartments was quantified in five randomly selected photomicrographs (taken using a ×40 objective). **c** Localization of CYTO-ID and Hoechst 33342 fluorescence dyes in IMR-32 cells was assessed using fluorescence microscope at 48 h. Scale bar 25 μm. C—control, PBS-treated cells; 14G2a—the 14G2a mAb-treated cells; CQ—chloroquine-treated cells; PC—positive control, cells grown in amino acids-free medium for 24 h. P-values for *t* test were as follow: *p < 0.05

We also measured fluorescence of the CYTO-ID dye to estimate autophagic flux in IMR-32 cells treated with the 14G2a mAb and/or CQ (Fig. 2a). Application of this dye was reported to be effective in labeling autophagic compartments with minimal staining of lysosomes [28]. The 14G2a mAb treatment shows the induction of CYTO-ID-estimated autophagic flux in IMR-32 cells to approximately 1.3, measured as relative fluorescence intensity. While the 14G2a mAb and CQ combined treatment indicates that autophagic flux is affected in IMR-32 cells as the fluorescence intensity of CYTO-ID is significantly increased when treated with both agents (in range of 2.2–3.3 depending on incubation time), as compared to single agent-treated IMR-32 cells and to control. To further explore the ability of the 14G2a mAb to induce autophagy in IMR-32 cells, the autophagic compartments, a highly characteristic feature of autophagic cells, cells were stained with CYTO-ID dye and visualized by fluorescent microscopy. We observed only a slight non-significant increase in the CYTO-ID dye fluorescence signal in the 14G2a mAb treated cells to 1.2, as compared to the control cells (Fig. 2b). However, we demonstrated that the fluorescence signal of autophagic compartments was by far the greatest for the combination of the 14G2a mAb and CQ, as compared to the control cells. It was elevated to 2.0 for the combination treatment and exceeded the fluorescence signal observed for CQ-treated cells (1.8), providing that induction of autophagy is present in these cells. Autophagic compartments appeared as green fluorescent cytoplasmic vesicles, while nuclei were blue-stained with the Hoechst 33342 dye (Fig. 2c). The observed accumulation of autophagic compartments is one of the hallmark of autophagic response. On the contrary, monitoring of relative fluorescence intensity of CYTO-ID in time in CHP-134 cells by a microplate reader (Supplementary Fig. 2a) and detection of CYTO-ID fluorescence signal by fluorescence microscopy (Supplementary Fig. 2b, c) showed no advanced autophagy in the 14G2 mAb-treated cells, as compared to control cells.

### Monitoring of autophagosomes formation by electron microscopy in the 14G2a mAb-treated IMR-32 and CHP-134 cells

**Fig. 3:**
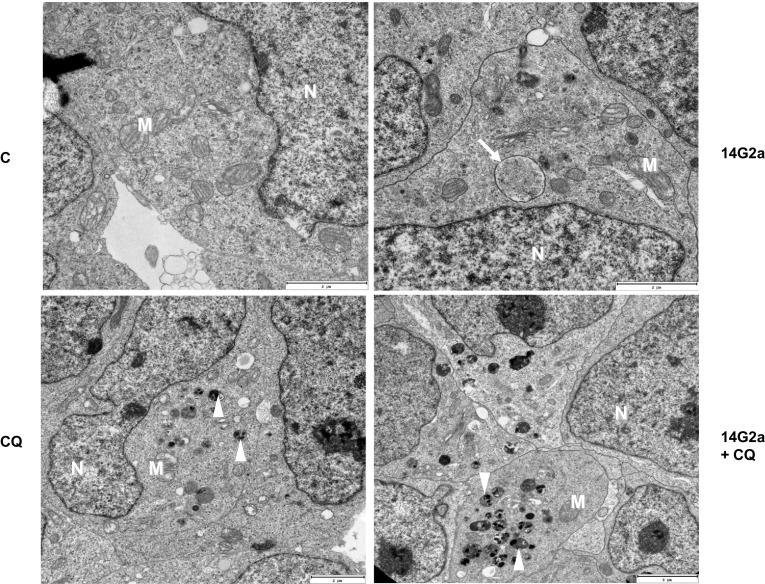
Assessment of autophagy features of the 14G2a-treated IMR-32 cells by TEM. IMR-32 cells were pre-treated with 10 μM CQ for 1.5 h and subsequently treated with the 14G2a mAb or PBS for 48 h, and visualized by TEM. The representative images account for exemplary photos of cells selected from at least 20 other photos of cells visualized under the microscope for each type of treatment. Autophagosomes (arrows) and residual bodies (arrowheads) are scattered along a cytoplasm. Scale bar 2 μm. N—nucleus, M—mitochondria. C—control, PBS-treated cells; 14G2a—the 14G2a mAb-treated cells; CQ—chloroquine-treated cells

Identification of autophagosomes/autolysosomes and monitoring of their number were done by TEM in the control, the 14G2a mAb-treated and the 14G2a and/or CQ-treated neuroblastoma IMR-32 and CHP-134 cells. At the ultrastructural level, an autophagosome is defined as a double-membraned structure containing undigested cytoplasmic contents, which has not yet fused with a lysosome. While, the autolysosome is a hybrid organelle generated by the fusion of an autophagosome and a lysosome, which has a single limiting membrane and contains cytoplasmic materials at various stages of degradation [29]. In the 14G2a mAb-treated IMR-32 cells, we observed a wide spectrum of cellular structures including autophagosomes that contain undigested cellular components, vesicles containing electron-dense undigested material or containing partially degraded granular cytoplasm indicative of autophagic activity (Fig. 3). Number of autophagosomes that appears in cytoplasm was juxtaposed in groups of the 14G2a mAb-treated and the control IMR-32 cells. We observed numerous and relatively few such structures in the respective groups of cells. Moreover, ultrastructural cell morphology shows the typical hallmarks of autophagy, especially in the 14G2a mAb-treated cells. There is no nuclear chromatin condensation and endoplasmic reticulum is swollen. Interestingly, after treatment with the CQ, a great number of residual bodies (telolysosomes) that contain material not digested by lysosomal enzymes, can be visualized in the cytoplasm of the IMR-32 cells. The percentage of these structures on average accounted for 5.1% of a cell cross-section. These structures were even more abundant in the 14G2a mAb and CQ-treated cells, where the percentage of these structures on average accounted for 10.4% of a cell cross-section. This, once again, corresponds to autophagy process being inhibited via CQ in the 14G2a mAb-treated IMR-32 cells. In CHP-134 cells, accumulation of insoluble, electron-dense aggregates, perhaps too large to be engulfed by autophagosomes or not recognized as material to be targeted for autophagic degradation, was also shown in the 14G2a mAb/CQ-treated cells, however to a lesser extent (Supplementary Fig. 3).

### Elucidation of autophagy role in the 14G2 mAb-induced cell death of IMR-32 cells

**Fig. 4:**
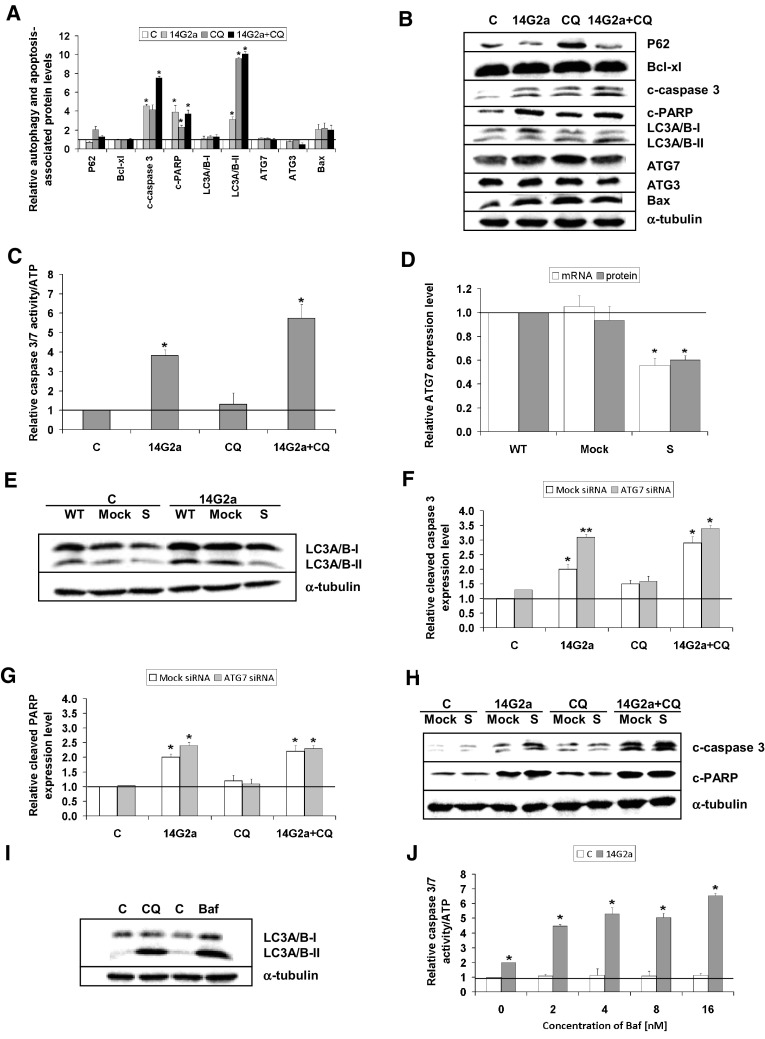
Effects of interfering with the 14G2a mAb-stimulated autophagy on the 14G2a/CQ-induced apoptosis. **a** Cells were pre-treated with 10 μM CQ for 1.5 h and subsequently treated with the 14G2a mAb or PBS (control) for 72 h. Relative autophagy and apoptosis-associated protein expression levels (P62, Bcl-xl, cleaved caspase 3, cleaved PARP, LC3A/B-I, LC3A/B-II, ATG7, ATG3, and Bax) were measured in control and the 14G2a mAb and/or CQ treated IMR-32 cells by western blot and normalized to α-tubulin. **b** Representative blots are presented. **c** Relative caspase 3/7 level was measured in control and 14G2a mAb and/or CQ-treated IMR-32 cells and was divided by ATP level signals of the respective groups of cells. **d** IMR-32 cells were transfected with *ATG7* siRNA (S) or control siRNA (Mock). The *ATG7* gene mRNA and ATG7 protein expression levels were analyzed at 24 h by RT-qPCR and western blot, respectively and compared to non-transfected cells (WT). *RPS13* cDNA and α-tubulin protein were used respectively, as the references. **e** IMR-32 cells were treated 24 h after siRNA transfection with the 14G2a mAb (40 μg/ml) or PBS (control) for 24 h. LC3A/B expression was analyzed by western blot, α-tubulin served as the loading control. **f** Relative expression levels of cleaved caspase 3 and **g** cleaved PARP were measured at 24 h after transfection in the 14G2a mAb, and/or CQ-treated *ATG7* silenced (S), the control cells (Mock). **h** Representative blots are presented. **i** LC3A/B expression was determined by western blot in IMR-32 cells treated with 10 μM CQ or 16 nM Baf for 24 h. **j** The relative caspase 3/7 level was measured in IMR-32 cells treated with 14G2a mAb (40 μg/ml), and/or indicated concentrations of Baf or its solvent—DMSO, and divided by ATP level signals of the respective groups of cells. C—control, solvent-treated cells (PBS for the 14G2a mAb, water for CQ or DMSO for Baf); 14G2a—the 14G2a mAb-treated cells; CQ—chloroquine-treated cells; Baf—bafilomycin-treated cells. P-values for *t* test were as follow: *p < 0.05, **p < 0.01

Our studies showed that the 14G2a mAb induces autophagy in IMR-32 neuroblastoma cells. Therefore, we wanted to determine the significance of autophagy in the 14G2a mAb-mediated cytotoxicity in IMR-32 cells. Autophagy process may act as a double edged sword by being either pro- or anti-tumor, depending upon a tumor type or a drug used. In order to define the role of the autophagy in the 14G2a mAb-cell killing, we asked whether disruption of the autophagic flux sensitizes IMR-32 neuroblastoma cells to the 14G2a mAb-induced cytotoxicity. To this end, we used the lysosomotropic agent—CQ, which inhibits autophagosome-lysosome fusion. We studied if CQ acts in concert with the 14G2a mAb to cause greater loss in cellular ATP level in IMR-32 neuroblastoma cells. Inhibition of autophagy by indicated concentrations of CQ (1.25–80 μM) potentiated cytotoxic effect of the 14G2a mAb, as compared to CQ used alone, as assessed by measuring cellular ATP content (Supplementary Fig. 4a). However, only for the range of CQ concentration (10–80 μM) used in combination with the 14G2a mAb, a visible decrease in the ATP level was observed, as compared to the 14G2a mAb used alone. Such an effect was not observed for lower concentration of CQ (1.25–5 μM) used in combination with the 14G2a mAb. In the presence of the 14G2a mAb and CQ, the IC50 value was 18.92 ± 0.99, which was lower than the IC50 value for CQ alone (22.62 ± 0.76). Similarly, simultaneous combination of the 14G2a mAb treatment and inhibition of autophagy by bafilomycin A1 (a specific inhibitor of the lysosomal proton pump, which inhibits autophagolysosome formation, abbreviated further to Baf), causes advanced cytotoxicity as compared to monotherapy with the Baf (Supplementary Fig. 4b). For the double-agent regimen, the IC50 value was 24.72 ± 5.02, which was higher than the IC50 value for Baf alone (20.07 ± 2.06). Most importantly, disruption of the autophagic flux by CQ sensitizes neuroblastoma cells to the 14G2a mAb-induced apoptosis, as assessed by an increase of cleaved-caspase 3, cleaved PARP, and pro-apoptotic Bax protein level signals to 7.5, 3.7 and 2.0, respectively (Fig. 4a, b). Apoptosis analyzed by measuring activity of caspase 3/7 is also significantly increased to 5.7 in IMR-32 cells treated with combination of the 14G2a mAb and CQ, as compared to both agents used alone and the control cells (Fig. 4c). Our previous studies showed that treatment with the 14G2a mAb downregulates the PI3K/AKT/mTOR pathway [30], and in this study we postulate that the 14G2a mAb triggers autophagy in IMR-32 cells. Since autophagy has been described as an adaptive stress response that promotes cell survival upon PI3K/mTOR inhibition [31], we further investigated the functional relevance of autophagy in the 14G2a mAb/CQ-mediated apoptosis in IMR-32 cells. To this end, we used two approaches to block autophagy. In the first approach, we inhibited autophagy at the early phase by siRNAs-mediated silencing of *ATG7* in IMR-32 cells. IMR-32 cells were transfected with *ATG7* siRNAs or control (Mock) siRNAs, and the *ATG7* gene mRNA as well as ATG7 protein expression were analyzed, as compared to the non-transfected (WT) cells. The *ATG7* gene mRNA expression and protein levels were significantly reduced at 24 h after transfection to 0.5 and 0.6, respectively (Fig. 4d). Furthermore, IMR-32 cells were treated 24 h after transfection with the 14G2a mAb (40 μg/ml) or PBS (control) for the next 24 h and LC3A/B expression was analyzed by western blot. The experiments showed that knockdown of *ATG7* resulted in decreased conversion of LC3A/B-I into LC3A/B-II form both in control and the 14G2a mAb-treated cells, demonstrating that *ATG7* silencing reduced basal and the 14G2a mAb-stimulated autophagy (Fig. 4e). The effect of *ATG7* silencing on apoptosis was concluded upon assessing cleaved caspase 3 and cleaved PARP levels. Measuring levels of cleaved caspase 3 and cleaved PARP and comparing it to the control (Mock) cells demonstrated no induction of apoptotic features in control and CQ-treated IMR-32 cells (Fig. 4f–h). However, for the 14G2a mAb-treated IMR-32 cells, we showed increase in cleaved caspase 3 and cleaved PARP in *ATG7*-silenced cells, as compared to Mock cells, indicating that this treatment rendered IMR-32 cells more prone to apoptotic death. Therefore, we can conclude that *ATG7* knockdown with siRNA increased the induction of apoptosis by monotherapy with the 14G2a mAb and to a smaller extent by combination treatment with the 14G2a mAb plus CQ. These results of treatment with the 14G2a mAb in conditions of *ATG7* silencing prove that inhibition of autophagy at the early stage acts in favor of the 14G2a mAb-induced apoptosis. The 14G2a mAb and CQ treatment as well as knockdown of *ATG7* showed greater effects on inducing apoptotic features, indicating that interfering with autophagy at an early step supports the 14G2a mAb-induced apoptosis. In the second approach we interfered with the late phase of autophagy using Baf. Baf was also similarly potent as compared to CQ to interrupt the autophagic flux as indicated by the accumulation of LC3A/B-II (Fig. 4i). Combined treatment with the 14G2a mAb (40 μg/ml) and Baf (at the final concentration of 16 nM) showed by far the greatest induction of apoptosis, that increased about threefold, as compared to monotherapy with the 14G2a mAb and about sixfold as compared to Baf used alone, analyzed by measuring caspase 3/7 activities (Fig. 4j). This set of results indicates that interfering with autophagy at a late step also augments the 14G2a mAb-induced apoptosis. Finally, we could conclude that inhibition of autophagy at both the early and the late stage is the primary mechanism of the CQ-mediated sensitization to the 14G2a-induced apoptosis.

### Assessing relevance of autophagy in non-apoptotic setting in CHP-134, LA-N-1 and LA-N-5 cells

Another interesting aspect which was explored, was significance of autophagy in neuroblastoma cell lines which have shown no apoptotic characteristics upon the 14G2a mAb treatment. We have previously shown that the 14G2a mAb causes cell death with partial characteristics of apoptosis in IMR-32 cells and no evident features of apoptosis in CHP-134, LA-N-1 and LA-N-5 cells, indicating disparate mechanisms of the 14G2a mAb-induced cell killing in different cell lines [11]. Our results showed also that the 14G2a mAb does not induce autophagy in CHP-134 beyond basal level. Firstly, we aimed to clarify how inhibition of autophagy at a constitutive level may influence cytotoxic effect exerted by the 14G2a mAb on CHP-134, LA-N-1 and LA-N-5 cells, in which apparent apoptotic features were not stated. Therefore, we asked if the inhibition of autophagy by CQ and Baf inhibitors, enhances the 14G2a mAb-induced cytotoxicity. Combined treatment with the 14G2a mAb (40 μg/ml) and indicated concentrations of CQ (2.5–10 μM) proved to have no greater effect on cellular ATP level, as compared to the 14G2a mAb used alone in CHP-134 (Supplementary Fig. 4c), LA-N-1 cells (Supplementary Fig. 4e) and LA-N-5 (Supplementary Fig. 4g). Neither simultaneous treatment with the 14G2a mAb and inhibition of autophagolysosome formation by Baf in a range of tested concentrations (2–8 nM) resulted in enhanced tumor cells-killing, as compared to the 14G2a mAb treatment alone, as determined by measuring cellular ATP content in CHP-134 (Supplementary Fig. 4d), LA-N-1 (Supplementary Fig. 4f) and LA-N-5 (Supplementary Fig. 4h). Only for 20 μM concentration of CQ used in combination with the 14G2a mAb, visible decrease in ATP level was observed in all neuroblastoma line tested, as compared to the 14G2a mAb used alone. However, in CHP-134 and LA-N-5 cells, treated with the 14G2a mAb and CQ, reduction of ATP level was measured, as compared to monotherapy with CQ, showing greater potential of autophagy inhibition for the optimal 14G2a mAb-induced tumor cells-killing. Similar effects in potentiating cytotoxicity against tested neuroblastoma cells were also noted for the 14G2a mAb and Baf-combined treatment, as compared to Baf-treated cells (used in concentration of 16 μM) in CHP-134 cells and LA-N-5.

### Characterization of the PHLDA1 protein role in regulation of autophagy and apoptosis in CHP-134 cells upon the 14G2a mAb treatment

*PHLDA1*/*TDAG51* (pleckstrin homology-like domain family A member 1/T cell death-associated gene 51) was first identified in murine T lymphocytes where it is required for activation of induced cell death [32]. The gene encodes a 401-amino acid protein that contains a central pleckstrin homology domain common to proteins involved in intracellular signaling, or found in constituents of the cytoskeleton. The protein is ubiquitously expressed in a wide range of normal and cancer tissues. Its function depends on cell type and cellular environment, becoming either proapoptotic or prosurvival [33]. PHLDA1 expression is induced by a variety of external stimuli, and there is evidence showing that it might act as a mediator of both autophagy and apoptosis, although the exact biochemical and biological function of PHLDA1 is still unclear. The *PHLDA1* gene was found to be up-regulated in both autophagy and apoptosis processes induced by rapamycin and silencing of this gene resulted in a reduction of these activities in T-47D breast carcinoma cells [34]. This observation clearly indicates that PHLDA1 positively regulates both apoptosis and autophagy pathways.

**Fig. 5:**
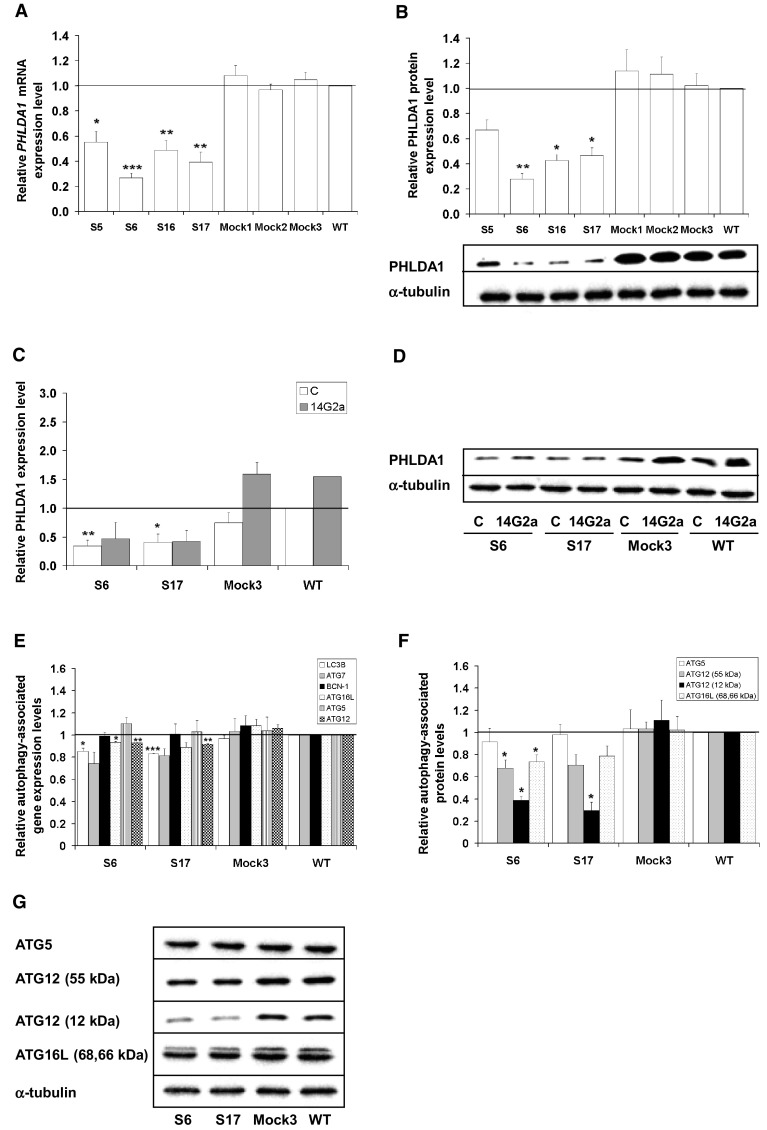
PHLDA1 downregulation in CHP-134 cells and its consequences on autophagy-associated transcripts and proteins. **a** The mRNA levels of *PHLDA1* gene were measured by RT-qPCR in *PHLDA1*-silenced (S5, S6, S16, and S17), Mock1–3, and WT cells. *RPS13* cDNA was used as the reference. The *PHLDA1* gene mRNA expression in WT cells equals 1 (black baseline). **b** The PHLDA1 protein expression was measured in *PHLDA1*-silenced (S5, S6, S16, and S17), Mock1–3 and WT cells by western blot at 48 h and normalized to α-tubulin. Below the chart representative blots are presented. **c** Relative expression of the PHLDA1 protein in PBS-treated and in the 14G2a mAb-treated selected *PHLDA1*-silenced clones (S6, S17), Mock3 and WT cells. Mean values for the 14G2a mAb-treated CHP-134 are presented as grey bars and calculated versus control values (white bars) for WT cells (black baseline). **d** Representative immunoblots are presented. C—control, PBS-treated cells; 14G2a—the 14G2a mAb-treated cells. **e** The mRNA levels of the *LC3B, ATG7, BCN-1, ATG16L, ATG5* and *ATG12* genes were measured by RT-qPCR in *PHLDA1*-silenced (S6 and S17), Mock3, and WT cells at 48 h. **f** The ATG5, ATG12, and ATG16L protein expression levels were measured in *PHLDA1*-silenced (S6 and S17), Mock3 and WT cells at 48 h by western blot. **g** Representative immunoblots are presented. P-values for *t* test were as follow: *p < 0.05, **p < 0.01, ***p < 0.001

**Fig. 6:**
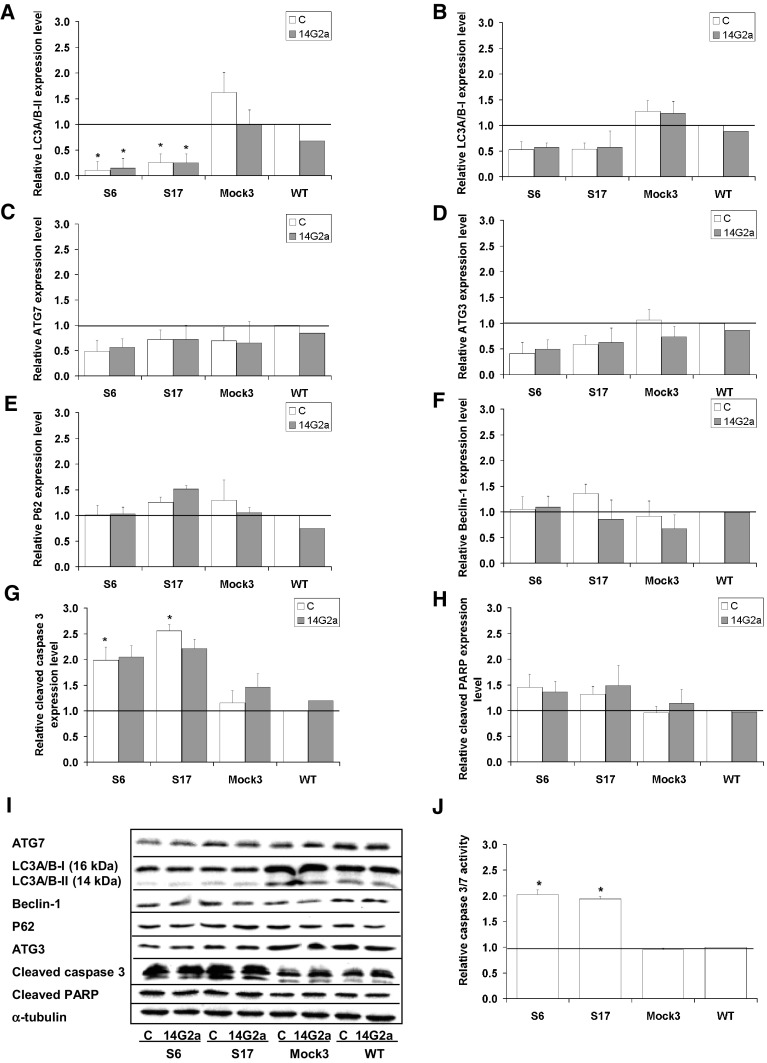
Effects of stable silencing of the *PHLDA1* gene on expression of autophagy and apoptosis-associated proteins in the 14G2a mAb-treated CHP-134 clones. Relative expression of LC3A/B-II (**a**), LC3A/B-I (**b**), ATG7 (**c**), ATG3 (**d**), P62 (**e**), Beclin-1 (**f**), cleaved caspase 3 (**g**), and cleaved PARP (**h**) proteins was assessed in selected *PHLDA1*-silenced (S6, S17), Mock3, and WT cells. Mean values for the 14G2a mAb-treated CHP-134 are presented as grey bars and calculated versus control values (white bars) for WT cells (black baseline). **i** Representative immunoblots are presented. **j** Relative activities of caspase 3 and 7 were calculated as caspase 3 and 7 activities at day 3 after treatment and divided by caspase 3 and 7 activities at day 1 in *PHLDA1*-silenced (S6, S17), Mock3 cells, and calculated versus control values for WT cells (black baseline). P-values for *t* test were as follow: *p < 0.05

We have previously implicated that the PHLDA1 protein level is significantly increased after the 14G2a mAb treatment, as compared to the control IMR-32 cells [11]. In addition, we have previously shown that silencing of the *PHLDA1* gene results in induction of autophagic activity and inhibits apoptotic characteristics in IMR-32 cells when treated with the 14G2a mAb, thus contributing to apoptosis resistance [21]. To explain the observed difference between IMR-32 and CHP-134 in autophagy regulation, with no autophagy stimulation upon the 14G2a mAb treatment in the latter, we further elaborated on the potential role of PHLDA1 in autophagy modulation in the CHP-134 cell line. Our results along with findings of other groups prompted us to investigate the possible role of PHLDA1 as an autophagy modulator in neuroblastoma cells. To address a question if PHLDA1 positively modulates autophagy, we derived CHP-134 cells with stably silenced expression of the *PHLDA1* gene by a lentivirus vector-based RNAi approach. The expression of *PHLDA1* gene in four selected *PHLDA1*-silenced clones (S5, S6, S16, S17) ranged from 0.3 to 0.5, as assessed by qPCR, in comparison to control (Mock) and non-transduced (WT) cells, for which expression level equaled 1 (Fig. 5a). Consequently, the expression of the PHLDA1 protein was reduced in all four selected clones with signals ranging from 0.3 to 0.7 of WT, as assessed by western blot (Fig. 5b). Next, the expression of autophagy-associated proteins was screened in these clones (Supplementary Fig. 5a). We observed that LC3A/B-II level was markedly decreased in all four *PHLDA1*-silenced clones, which was accompanied by an evident increase in the P62 level, as compared to WT cells. This inverse correlation corresponds to autophagy process being inhibited upon silencing of the *PHLDA1* gene. Moreover, an increase in apoptosis-associated proteins i.e., cleaved caspase 3 and cleaved PARP was detected in the *PHLDA1*-silenced clones, as compared to Mock and WT cells (Supplementary Fig. 5b). Detection of autophagy and apoptosis markers allowed us to correlate both processes in the opposite manner in *PHLDA1*-silenced cells. More thorough analysis was carried out further in two selected *PHLDA1*-silenced clones, exhibiting the lowest *PHLDA1* expression level (S6 and S17), to assess the role of PHLDA1 in the settings of the 14G2a mAb treatment and the crosstalk between autophagy and apoptosis. Prior to this analysis, we verified that there is no difference in ATP levels between *PHLDA1-*silenced (S6 and S17), Mock3, and WT cells grown in culture for 48 h, so as the observed results could reflect the effects of particular treatment. We observed up-regulation of the PHLDA1 protein signal to approximately 1.6 at 48 h after the 14G2a mAb treatment in Mock and WT cells and no such a trend in *PHLDA1*-silenced clones (Fig. 5c, d). This attenuated effect of the 14G2a mAb on the PHLDA1 level may suggest the involvement of the PHLDA1 protein in the 14G2a mAb mode of treatment. We revealed that *PHLDA1* downregulation modulates levels of several autophagy-associated genes. In *PHLDA1*-silenced CHP-134 cells (S6 and S17), the *LC3B* and *ATG12* gene expression on mRNA levels were significantly reduced, as compared to Mock3 and WT cells (Fig. 5e). The *ATG7* and *ATG16L* mRNA expression levels were also decreased in S6 and S17 clones. However, the mRNA expression of *BCN-1* and *ATG5* genes was not changed, as compared to Mock3 and WT cells. Initially, this was followed by detecting the expression level of some autophagy-associated proteins i.e. ATG5, ATG12, and ATG16L in S6 and S17 clones of *PHLDA1*-silenced cells (Fig. 5f, g). While the ATG5 expression level remained unchanged, expression of ATG12 and ATG16L forms declined in both clones, as compared to Mock3 and WT cells. We further described the effect of *PHLDA1* downregulation on expression of molecules involved in autophagy process such as LC3A/B, Beclin-1, and ATG proteins in S6 and S17 clones (Fig. 6). Our results showed that PHLDA1 is a positive modulator of autophagy, as the expression signal of autophagy marker, the LC3A/B-II is decreased with statistical significance to 0.1 in S6 and 0.3 in S17 *PHLDA1*-silenced clones, as compared to the control (Mock3) and WT cells (Fig. 6a, i). Also signals of LC3A/B-I, ATG7, and ATG3 proteins are reduced in range of 0.4–0.6 (Fig. 6b–d, i), and only marginal changes or slight increase in P62 and Beclin-1 expression levels were observed (Fig. 6e, f, i). However, it must be emphasized that the role of PHLDA1 may not be limited only to autophagy modulation, as shown by detection of expression of the key apoptosis proteins in our model. Detection of apoptosis-associated molecules showed that the signal of expression of the cleaved caspase 3 increased twofold and 2.6-fold, respectively in S6 and S17 clones with statistical significance, as compared to Mock3 and WT cells (Fig. 6g, i). This change was parallel to an increase in signals of the cleaved PARP form in S6 and S17 clones to 1.5 and 1.3, respectively (Fig. 6h, i). Significant induction of caspase 3/7 activities to approximately 2 was also observed in both clones, as compared to Mock3 and WT cells (Fig. 6j). Therefore, we confirmed that PHLDA1 protein is able to negatively regulate apoptosis in CHP-134. The presented results show that PHLDA1 is involved in regulation of autophagy- and apoptosis-associated proteins and both processes exist in a mutually exclusive manner in *PHLDA1*-silenced CHP-134 cells.

### Autophagy detection in MK-5108-treated IMR-32 cells

**Fig. 7:**
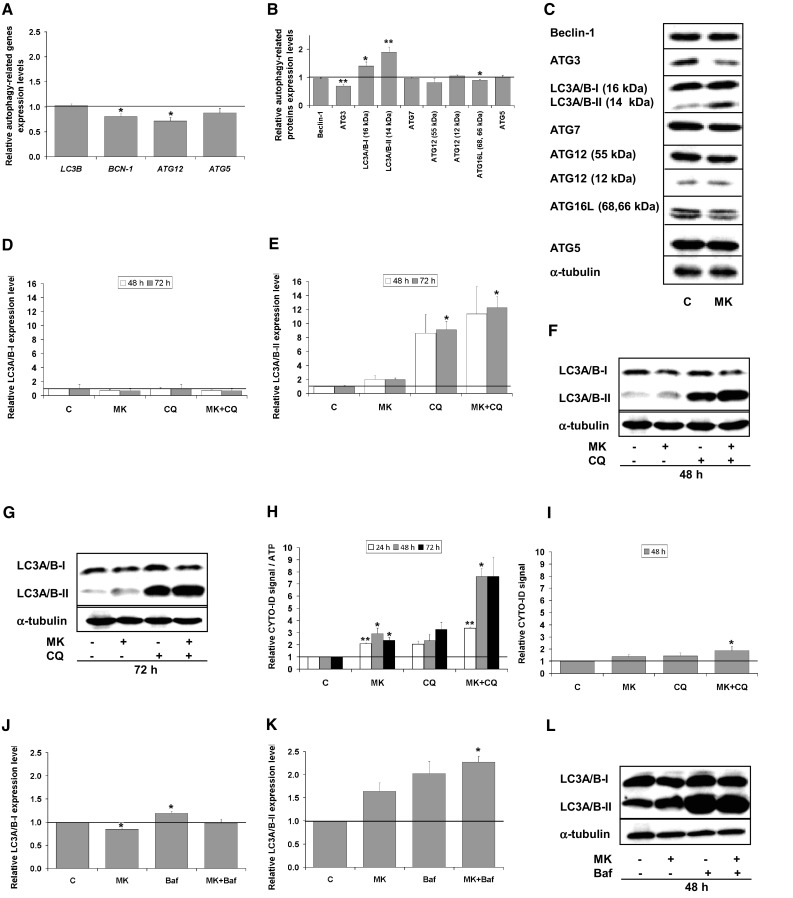
Autophagy in MK-5108-treated IMR-32 cells. **a** Relative gene expression level of *LC3B, BCN-1, ATG12*, and *ATG5* in MK-5108-treated IMR-32 cells (0.1 μM) as compared to the control (DMSO-treated cells) assessed at 48 h by RT-qPCR. *RPS13* cDNA was used as the reference. **b** Relative protein expression level of Beclin-1, ATG3, LC3A/B-I, LC3A/B-II, ATG7, ATG12, ATG16L, and ATG5 was measured at 48 h by western blot and normalized to α-tubulin. Expression of proteins and their respective genes in control cells equals 1 (black baseline). **c** Representative immunoblots are presented. **d** Relative LC3A/B-I expression level affected by MK-5108 or/and CQ treatment assessed by western blot. IMR-32 cells were pre-treated with 10 μM CQ for 1.5 h and subsequently treated with 0.1 μM MK-5108 or DMSO (control, inhibitor solvent) for 48 and 72 h. **e** LC3A/B-II—estimated autophagy flux affected by MK-5108 or/and CQ treatment assessed by western blot. **f, g** Representative immunoblots are presented. **h** CYTO-ID—estimated autophagy flux in IMR-32 cells. Relative mean fluorescence intensity of the CYTO-ID dye was measured at 24, 48 h and 72 h using a microplate reader and divided by the ATP level signals of the respective groups of cells. **i** Relative mean fluorescence intensity of the CYTO-ID-stained autophagic compartments was quantified in five randomly selected photomicrographs (taken using ×40 objective). Relative LC3A/B-I (**j**) and LC3A/B-II (**k**) expression levels affected by MK-5108 or/and Baf treatment assessed by western blot. IMR-32 cells were pre-treated with 16 nM Baf for 1.5 h and subsequently treated with 0.1 μM MK-5108 or DMSO for 48 h. **l** Representative immunoblots are presented. C—control, DMSO-treated cells; MK—MK-5108-treated cells; CQ—chloroquine-treated cells; Baf—bafilomycin A1-treated cells. P-values for *t* test were as follow: *p < 0.05, **p < 0.01

**Fig. 8:**
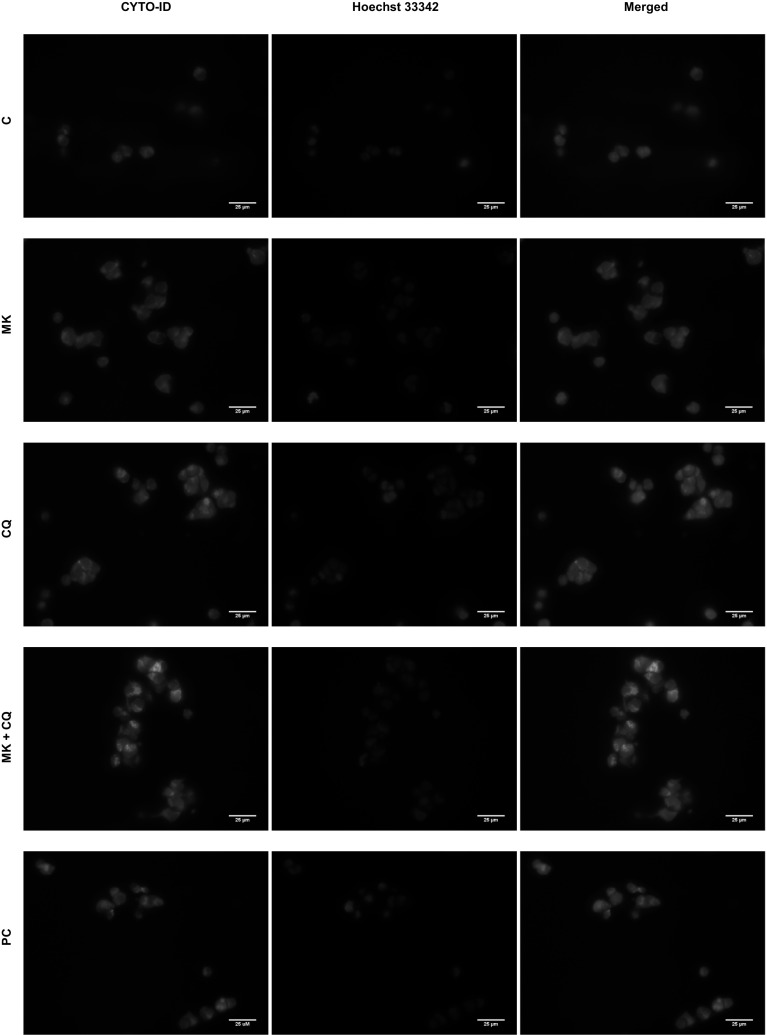
Monitoring of CYTO-ID dye fluorescence signal in autophagy compartments in IMR-32 cells. Localization of the CYTO-ID and Hoechst 33342 fluorescence dyes in IMR-32 cells was assessed using fluorescence microscope. IMR-32 cells were pre-treated with 10 μM CQ for 1.5 h and subsequently treated with 0.1 μM MK-5108 or DMSO for 48 h. The cells were then stained with CYTO-ID and Hoechst 33342, fixed, and visualized under fluorescence microscope. Relative mean fluorescence intensity of CYTO-ID-stained autophagic compartments was quantified in five randomly selected photomicrographs (taken using a ×40 objective). Scale bar 25 μm. C—control, DMSO-treated cells, MK—MK-5108-treated IMR-32 cells, CQ—chloroquine-treated IMR-32 cells, PC—positive control, IMR-32 cells grown in amino acids-free medium for 24 h

Previously we have shown that the aurora A kinase small-molecule, specific inhibitor, MK-5108, combined with the GD2 specific 14G2a mAb significantly enhances cytotoxic effect against IMR-32 and CHP-134 cell cultures [11]. Therefore, we also investigated the potential of MK-5108 inhibitor in the regulation of autophagy in IMR-32 neuroblastoma cells. We measured relative expression level of autophagy-associated genes in the MK-5108-treated IMR-32 cells at 48 h (Fig. 7a). We observed significant inhibition of the *BCN-1* and *ATG12* genes to 0.8 and 0.7, respectively and no changes in expression levels of *LC3B* and *ATG5* genes. Importantly, MK-5108 treatment significantly induced the expression of the crucial autophagy marker (LC3A/B), as demonstrated here by up-regulation of the signal of the converted, autophagosome-associated LC3A/B-II form to 1.9 (Fig. 7b, c). We monitored autophagic flux by assessing the relative LC3A/B-II expression level measured by western blot (Fig. 7e–g). In fact, we observed LC3A/B-II estimated autophagic flux induced to approximately 2 by MK-5108 at 48 and 72 h, as compared to control cells. Next, IMR-32 cells were pre-treated with CQ and subsequently treated with MK-5108 or DMSO. We observed LC3A/B-II estimated autophagic flux affected by the CQ and MK-5108 combined treatment, as demonstrated by the further increase in the LC3A/B-II level to 11.4 (48 h) and 12.3 (72 h), that is prevented from degradation in autophagolysosomes by CQ (Fig. 7e–g). We also measured the CYTO-ID dye-estimated autophagic flux in IMR-32 cells (Fig. 7h). MK-5108 and CQ combined treatment indicates that autophagic flux is affected in IMR-32 cells as the fluorescence intensity of CYTO-ID is significantly increased to 7.6 when treated with both agents for 48 h, as compared to control or single agent-treated IMR-32 cells (Fig. 7h). We also checked LC3A/B-II-estimated autophagy after treatment with MK-5108 and Baf or both agents used alone (Fig. 7k, l). The use of such combination confirmed autophagy induction in MK-5108-treated IMR-32 cells, as the signal of LC3A/B-II form rose further to 2.3 in case of double treatment, as compared to MK-5108 (1.6) and Baf (2.0) used alone. Then, the cells were stained with CYTO-ID and Hoechst 33342, fixed and visualized under fluorescence microscope (Fig. 8). We observed by far the greatest CYTO-ID fluorescence signal for combination of MK-5108 and CQ. All the results presented so far provide first-hand evidence that MK-5108 inhibitor induces autophagy process in IMR-32 cells. Similar set of experiments was performed for CHP-134 cells (Supplementary Fig. 6a–i). We were able to state that also in CHP-134 cell line, MK-5108 inhibitor stimulates autophagic flux, as demonstrated by increase in LC3A/B-II protein expression level signals to 1.4 (at 48 h) and 2.0 (at 72 h) (Supplementary Fig. 6e). While addition of CQ in combination with MK-5108 inhibitor, significantly increased LC3A/B-II level signals to further extent (3.4 for 48 h and 4.6 for 72 h), as compared to both agents used alone (Supplementary Fig. 6e, g, h). These results were further supported by evidence that P62 autophagic marker protein is degraded at 72 h in MK-5108-treated cells and the signal of the expression level of P62 was markedly decreased to 1.6 in MK-5108 and CQ-treated cells, as compared to CQ monotherapy (1.8), indicating an actual induction of autophagy in CHP-134 cells (Supplementary Fig. 6f, h). To further confirm these results, the autophagic compartments were stained with the CYTO-ID dye and visualized by fluorescent microscopy (Supplementary Fig. 6i). We observed an increase in the CYTO-ID dye fluorescence signal in MK-5108 treated cells to 1.3, as compared to the control cells. Furthermore, we demonstrated that the fluorescence signal of autophagic compartments was by far the greatest for the combination of MK-5108 and CQ, as compared to the control cells. It was elevated to 2.5 for the combination treatment and exceeded the fluorescence signal observed for CQ-treated cells (2.0), providing that induction of autophagy is present in these cells.

## Discussion

The role of autophagy and its regulation in cancer cells continues to emerge. Therefore, an increased understanding of autophagy in neuroblastoma is important for its optimal exploitation for therapeutic advantage. Although important strides have been made, several key issues remain unresolved, including how autophagy is regulated in neuroblastoma tumor cells, the interplay between autophagy and apoptosis, and the specific mechanism by which autophagy may confer treatment resistance. The purpose of this study was to further investigate the molecular and cellular mechanisms of the cytotoxic effect of the 14G2a mAb on human neuroblastoma cell survival in vitro, especially in the context of autophagy regulation. We aimed to establish how treatment with the 14G2a mAb affects the functional status of autophagy in IMR-32 and CHP-134 cells.

Many proteins and molecular pathways, deregulated upon the anti-GD2 ganglioside 14G2a mAb treatment, such as inhibition of PI3K/AKT/mTOR signaling [30], suggested the possible induction of autophagy in IMR-32 and CHP-134 cells. Several experimental data gathered by us in this study demonstrated that autophagy is induced in response to the 14G2a mAb treatment in IMR-32 neuroblastoma cell line and therefore accounts for a crucial process, which may, next to apoptosis, dictate cell fate. However, the mechanism underlying activation of autophagy by binding the 14G2a mAb to GD2 ganglioside still remains unresolved. Only recently, reports are emerging that a glycosfingolipid—GD3 ganglioside, component of cell membranes, is involved in autophagosome formation and maturation in human and murine primary fibroblasts [35]. The results suggest that gangliosides through their interaction with autophagy-associated molecules, could be recruited to autophagosomes and contribute to morphogenic remodeling of autolysosomes. Furthermore, current literature supports the understanding that ceramide-induced autophagy functions to promote cell death, either through the induction of autophagy or by “switching off” autophagy and inducing apoptosis through the CAPN/calpin-mediated cleavage of ATG5 and/or DISC formation [36–38]. We have previously shown that chloroquine, an inhibitor of lysosomal degradation, did not rescue IMR-32 cells from antibody-induced cell death suggesting lack of ceramide involvement in the observed effect of the 14G2a mAb [6].

To meet expectations of proposing more effective immunotherapeutic strategy of neuroblastoma, we studied the role of autophagy process in the 14G2a mAb-treated IMR-32 neuroblastoma cells. We were able to show that interfering with autophagy at an early step by *ATG* silencing and a late step by using bafilomycin A1, augments the 14G2a mAb-induced apoptosis in IMR-32 cells. Our next goal was to define the role of the autophagy process in the 14G2a mAb-induced neuroblastoma cell killing, that may be either pro- or anti-tumor depending upon a cell line or a drug used. Hence, autophagy inhibitors such as chloroquine or bafilomycin A1 were tested to find the most effective combinatorial treatment with the 14G2a mAb. Combined treatment of IMR-32, CHP-134 and LAN-5 cells with the 14G2a mAb and either CQ or Baf showed potentiating of cytotoxic effects as compared to monotherapy with CQ or Baf, however in all cell lines tested, the combination of the 14G2a mAb and CQ or Baf was no more potent than the 14G2a mAb used alone for majority of concentrations used. It needs to be emphasized that effects of autophagy inhibition may be influenced by a few factors including the differences in an autophagy inhibitor, type of target cells, and/or degree of damage on target cells. Therefore, other specific inhibitors of autophagy such as 3-methyladenine (3-MA) and NH_4_Cl need to be tested in future and on other neuroblastoma cell lines. It is likely that these studies allow for better understanding of the mechanisms of the interplay between autophagy inhibitors and the 14G2a mAb that dictate a fate of neuroblastoma cells.

Previously we have shown that IMR-32 cells in response to the 14G2a mAb and the aurora A kinase specific inhibitor, MK-5108, undergo intensified cell death as compared to MK-5108 used alone [11]. In this study, we also presented evidence that MK-5108 induces autophagy process in IMR-32 and CHP-134 neuroblastoma cells. Recent findings show that in breast cancer depletion of aurora A by siRNA or chemical inhibition of aurora A by the small molecule VX-680 increased both the level of LC3-II and the number of autophagosomes. Conversely, overexpression of aurora A inhibited autophagy, as assessed by decreased LC3-II levels [39]. Moreover, the reports are emerging that another selective aurora A kinase inhibitor, Alisertib (ALS), exerts pro-autophagic effects on A375 and SK-MEL-5 melanoma cells by inhibiting P38 MAPK signaling. SB202190, a P38 MAPK-selective inhibitor, enhanced ALS-induced autophagy in both cell lines [40]. Moreover, ALS inhibitor was shown to induce autophagy in MCF7 and MDA-MB-231 cells via P38 MAPK [41]. Furthermore, entrectinib, small molecule inhibitor of major neuroblastoma pathogenic marker—anaplastic lymphoma kinase (ALK), exerts pro-autophagic effects on SH-SY5Y^F1174L^ neuroblastoma cells [42]. Abrogation of autophagy by chloroquine significantly increased the toxicity of entrectinib, as confirmed by enhanced death rate in SH-SY5Y^F1174L^ cells.

Our study also addressed a question if autophagy process may participate in the 14G2a-mediated cell death, manifesting itself especially in cells, in which apoptosis is not present. Therefore, we assessed the relevance of autophagy in non-apoptotic setting in CHP-134 cells. On the basis of various published findings and a set of our experiments, it was fairly justifiable to anticipate induction of autophagy in the 14G2a mAb-treated CHP-134 cells. However, this scenario was ruled out as autophagy is not enhanced beyond constitutive level after the 14G2a mAb treatment of CHP-134 cells. There are several findings showing that enhancement of autophagy may potentiate cell death in some models. Inducing autophagy with its activators may be alternative strategy worth exploring in CHP-134 cells. Findings described in [31] show that induction of autophagy can accelerate cytotoxic effects of chLym-1, a chimeric anti-human HLA-DR monoclonal antibody on Raji lymphoma cells. It was found that the viability of Raji cells treated with the chLym-1 and an autophagy activator, rapamycin, was significantly inhibited, as compared with that of the Raji cells treated with chLym-1 alone. While cells treated with chLym-1 in combination with autophagy inhibitors 3-MA and NH_4_Cl showed a significant rescue of cell viability after 48 h of co-incubation, 3-MA and NH_4_Cl alone have no significant effect on viability of Raji cells [31]. Other studies also revealed that inhibition of mTOR complex and enhancement of autophagy is involved in growth inhibition, apoptotic cell death in lymphoma cells, and may correlate with greater clinical outcome, suggesting a tumor-suppression role of autophagy in non-Hodgkin’s lymphoma treatment [43].

Lysosomal cell death, which could be yet another form of cell death involved in the observed effect of the 14G2a mAb, needs to be examined. It is initiated by lysosomal membrane permeabilization (LMP), which leads to the release of cathepsins and other hydrolases from the lysosomal lumen to the cytosol [44]. Depending on the extent of LMP and the cell type, LMP can result in apoptosis featuring caspase activation and mitochondrial outer membrane permeabilization or necrosis-like programmed cell death [45]. There are various stimuli that can trigger LMP, e.g., lysosomotropic compounds with detergent-like activity such as chloroquine [46]. LMP type of cell death will be further studied especially in apoptotic and non-apoptotic settings.

Interestingly, the evidence appeared on how the PHLDA1 protein might act as a mediator of both autophagy and apoptosis. The *PHLDA1* gene was reported to be up-regulated in both autophagy and apoptosis and silencing of this gene was found to reduce both activities, strongly suggesting that PHLDA1 mediates and positively regulates both autophagy and apoptosis pathways in T-47D breast carcinoma cells [34]. Therefore, we aimed to determine autophagy and apoptosis-linked biological function of PHLDA1 in CHP-134 cells. We followed *PHLDA1*-silencing approach in CHP-134 cells to explain molecular relationships and the circumstances that potentially could dictate the choice between apoptosis and autophagy in CHP-134 cells. Our results showed that PHLDA1 is a positive modulator of autophagy and both processes exist in a mutually exclusive manner in the *PHLDA1*-silenced CHP-134 cells. These results are contrary to our previous results in IMR-32 cells, where the function of PHLDA1 pointed to inhibition of autophagosomes formation and higher susceptibility to apoptosis [21]. Further studies are warranted to determine the perplexing role of PHLDA1 which, as it is implicated in many studies, relies on cell-specific environment [33].

It is still not clear whether and how autophagy may influence response of cancer cells to therapeutic agents. There is only little evidence that immunological mechanisms (ADCC, CDC) and apoptosis process induced by different chemotherapeutics are accompanied by autophagy process in cancer cells [31]. This broader context of interpretation of cell death mechanisms should be thoroughly considered to improve the efficacy of drugs used.

The need for defining optimal strategies to modulate autophagy for therapeutic advantage is still in its infancy. The combination of immune-based drugs i.e., mAb with autophagy modulators can broaden the treatment options of neuroblastoma. Targeting various molecular processes and pathways, instead of one, with drug combinations represents a new challenge in the coming decade but is of great promise. Innovative treatment strategies based on a better understanding of the crucial biological processes and pathways responsible for neuroblastoma initiation and progression display its potential in improvement of patients survival.

## Electronic supplementary material

Below is the link to the electronic supplementary material.

Supplementary material 1 (PPT 28552 KB)

Supplementary material 2 (DOC 47 KB)

## Abbreviations

FCS: Fetal calf serum
GD2: GD2 ganglioside
mAb: Monoclonal antibody
RT-qPCR: Reverse transcription and quantitative PCR
CQ: Chloroquine diphosphate salt
Baf: Bafilomycin A1
MK: MK-5108 inhibitor
