# Supplementary material for: GD2 ganglioside-binding antibody 14G2a and specific aurora A kinase inhibitor MK-5108 induce autophagy in IMR-32 neuroblastoma cells
Source: Apoptosis. 2018 Jul 19;23(9):492–511. doi: 10.1007/s10495-018-1472-9 (PMC6153511; doi:10.1007/s10495-018-1472-9)
Supplement: Supplementary file 1 — Supplementary material 1 (PPT 28552 KB) [file 10495_2018_1472_MOESM1_ESM.ppt]

## Slide 1
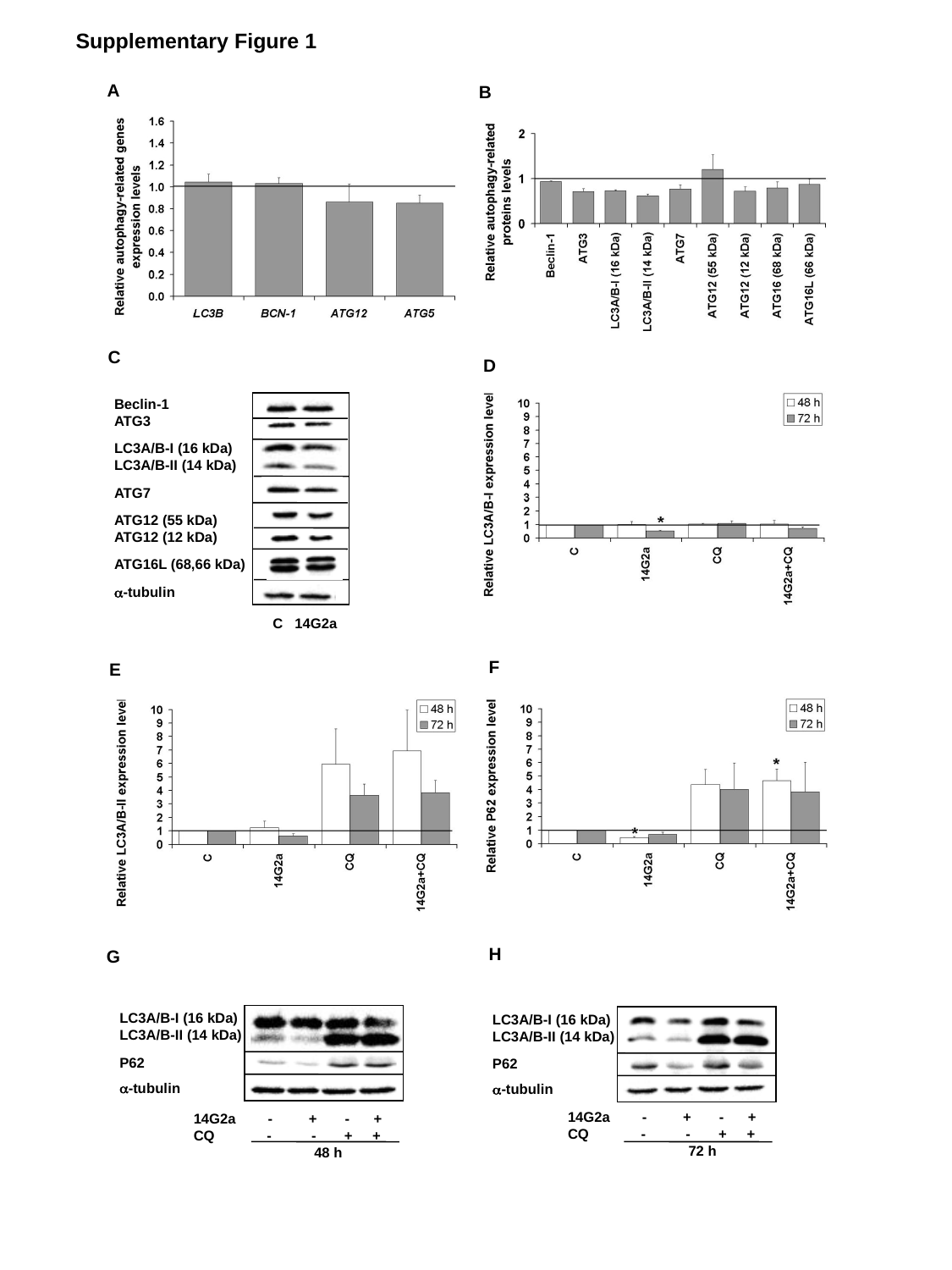

Supplementary Figure 1
A
B
C
D
Beclin-1
ATG3
LC3A/B-I (16 kDa)
LC3A/B-II (14 kDa)
ATG7
ATG12 (55 kDa)
ATG12 (12 kDa)
ATG16L (68,66 kDa)
α-tubulin
 C 14G2a
F
E
H
G
LC3A/B-I (16 kDa)
LC3A/B-II (14 kDa)
P62
α-tubulin
LC3A/B-I (16 kDa)
LC3A/B-II (14 kDa)
P62
α-tubulin
14G2a - + - +
CQ - - + +
 72 h
14G2a - + - +
CQ - - + +
 48 h

## Slide 2
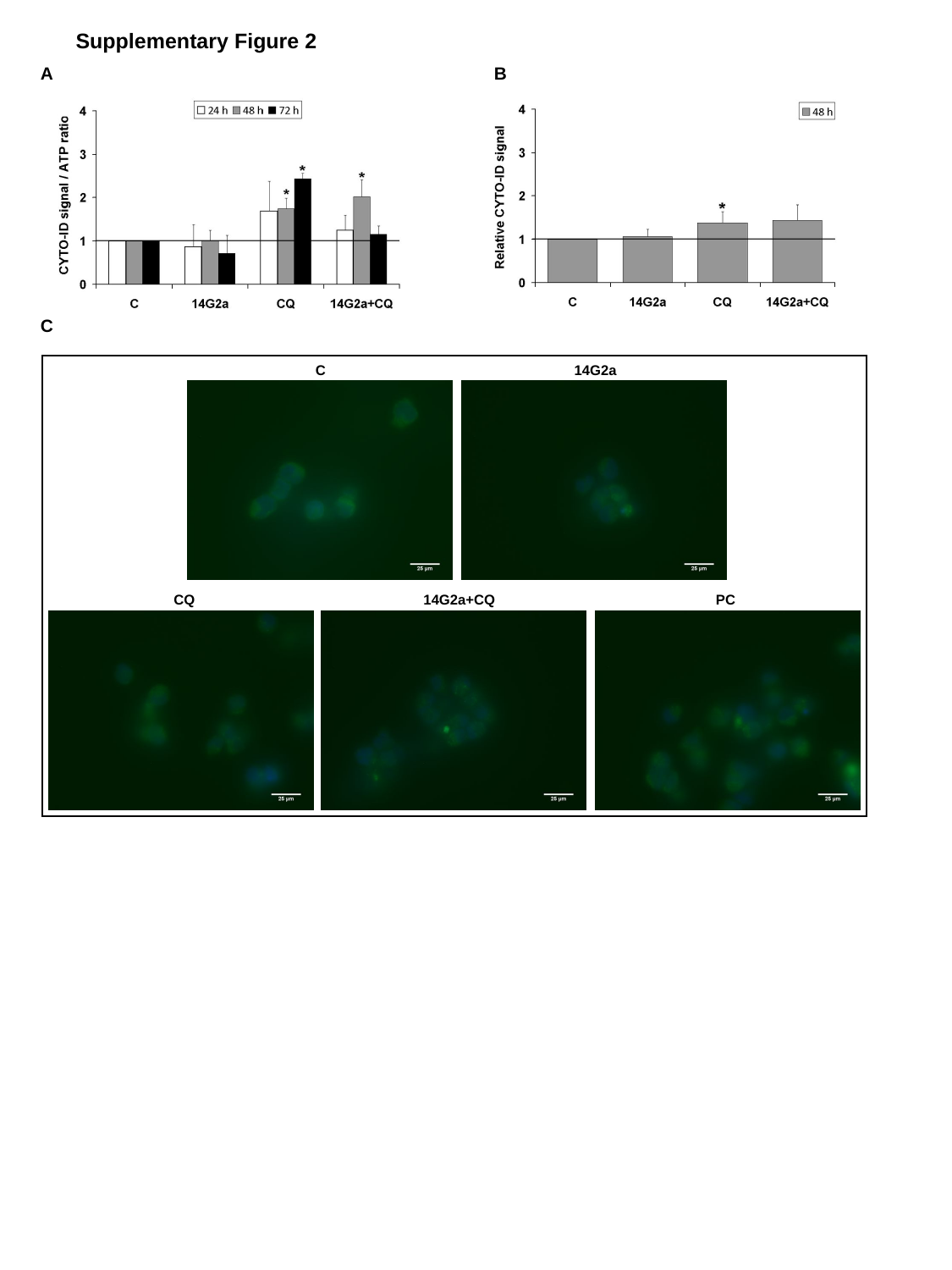

Supplementary Figure 2
A
B
C
 C 14G2a
CQ 14G2a+CQ PC

## Slide 3
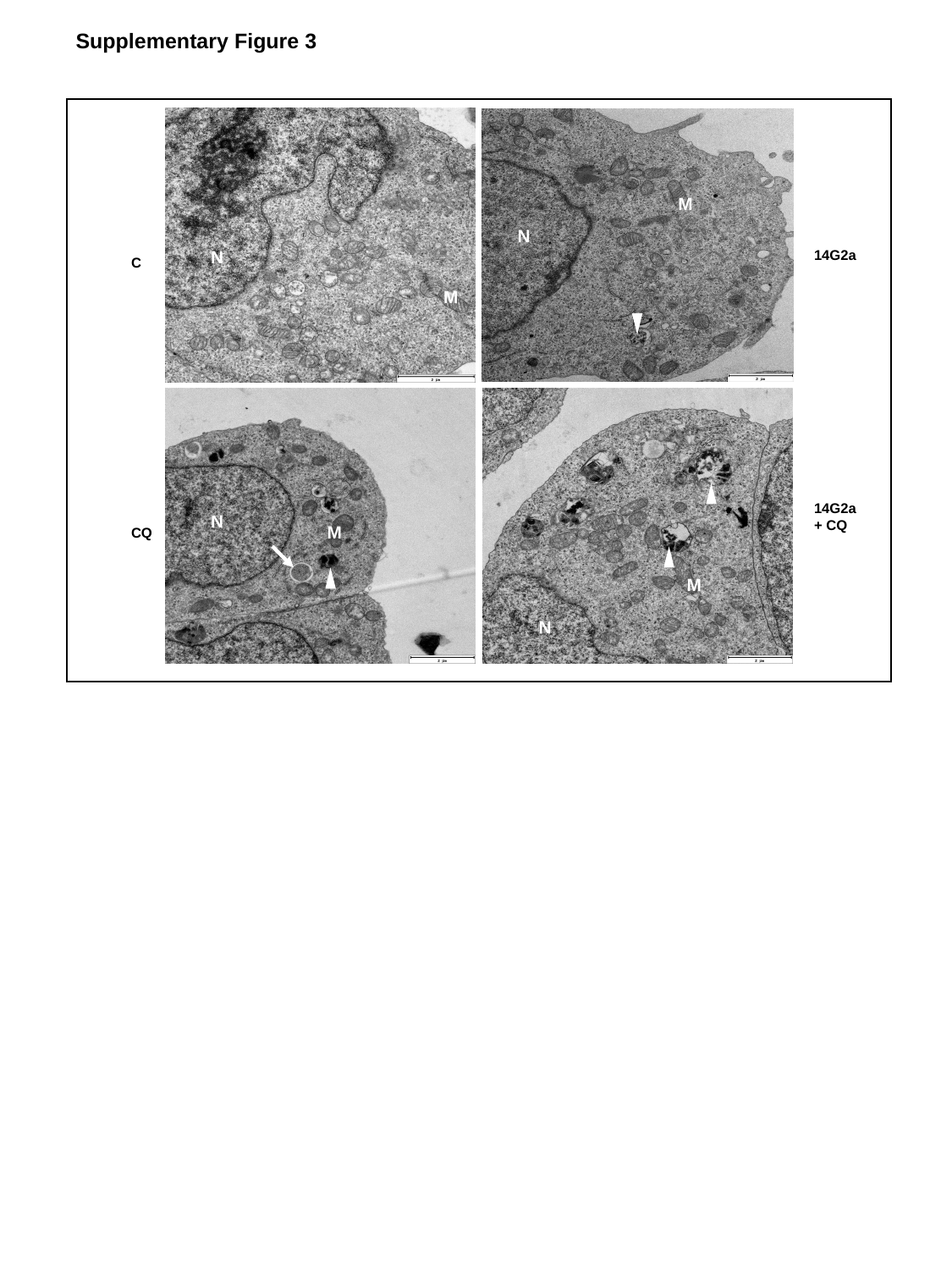

Supplementary Figure 3
M
N
N
14G2a
14G2a
+ CQ
C
CQ
M
N
M
M
N

## Slide 4
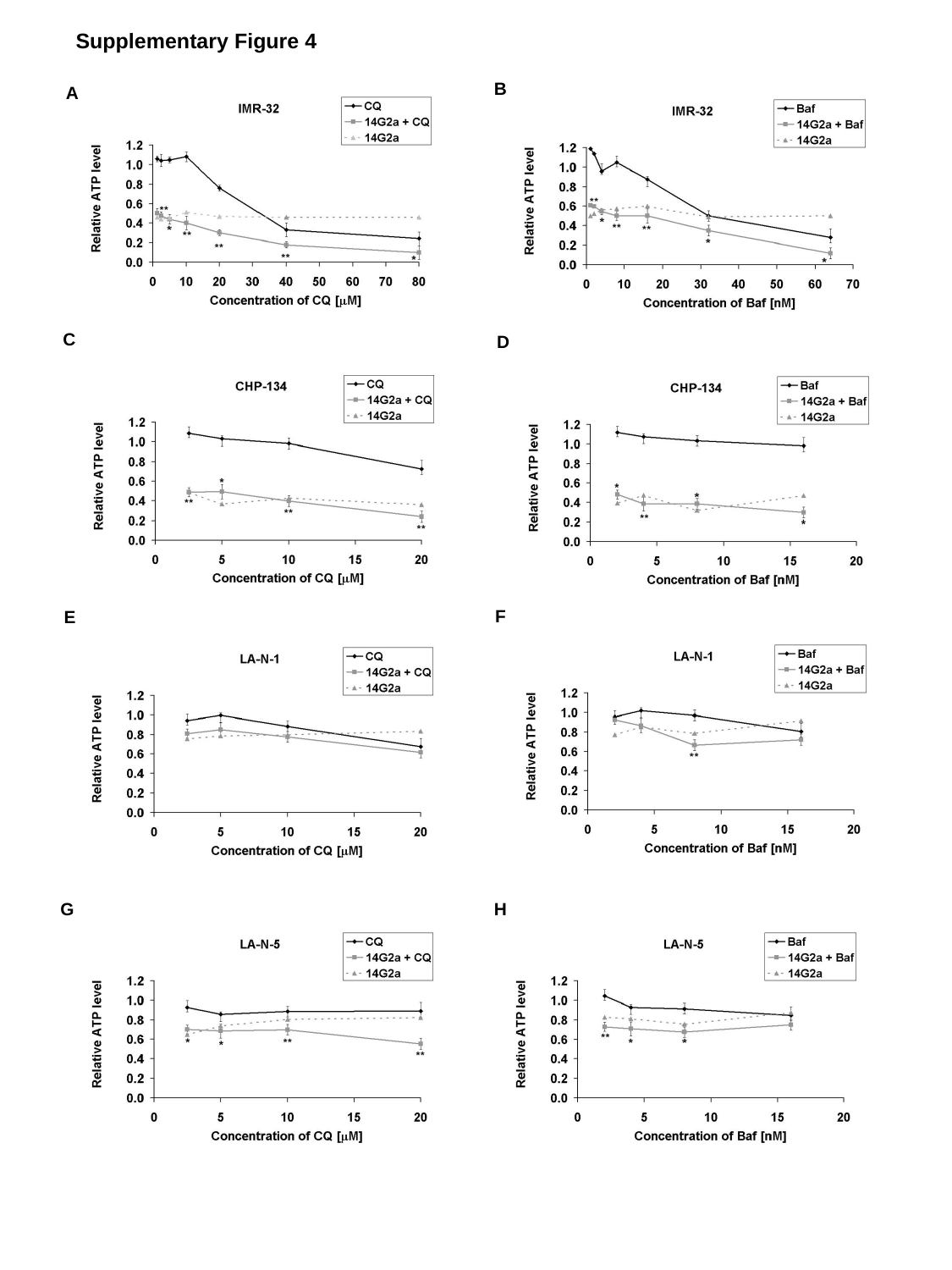

Supplementary Figure 4
B
A
C
D
F
E
G
H

## Slide 5
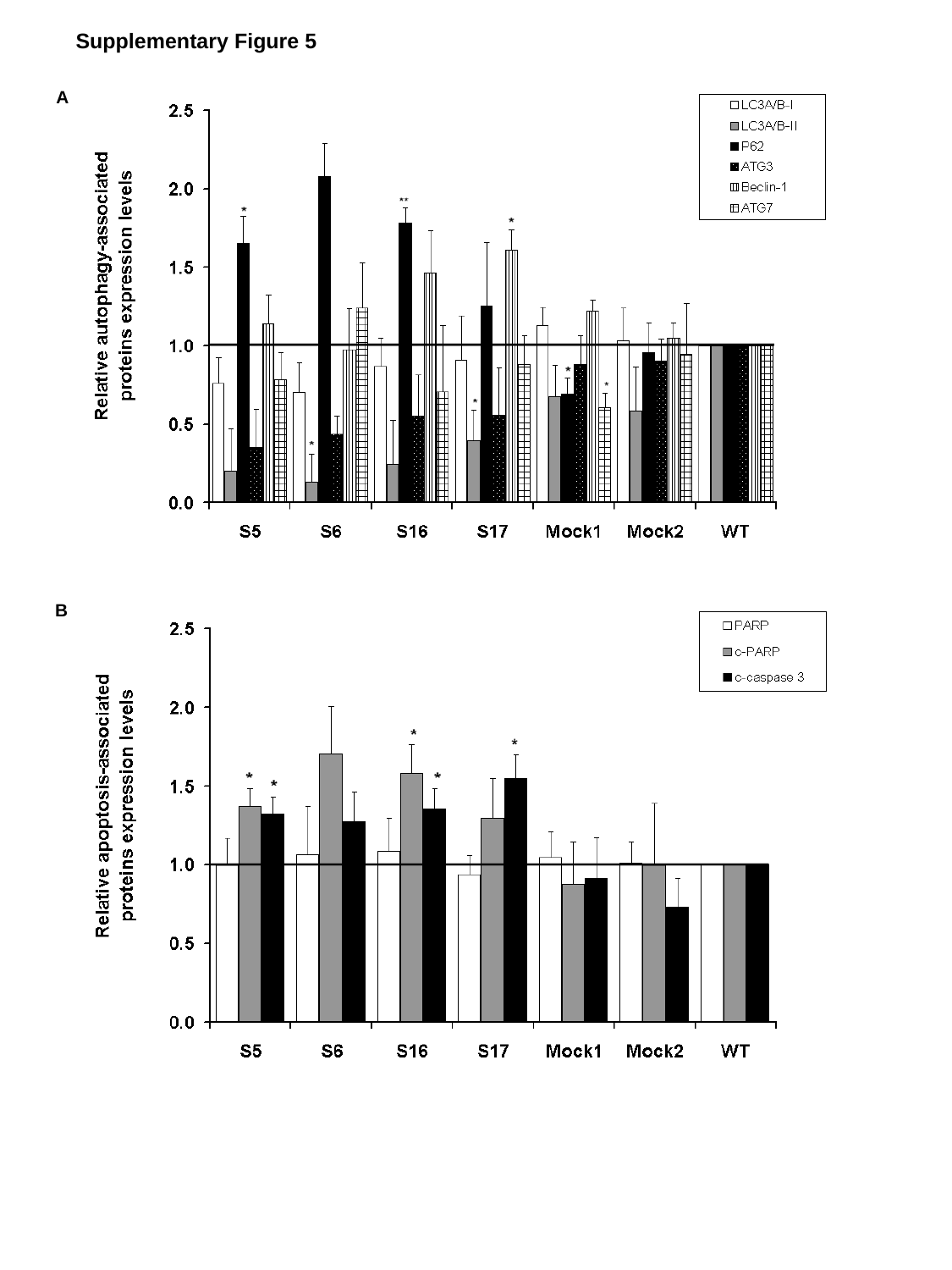

Supplementary Figure 5
A
B

## Slide 6
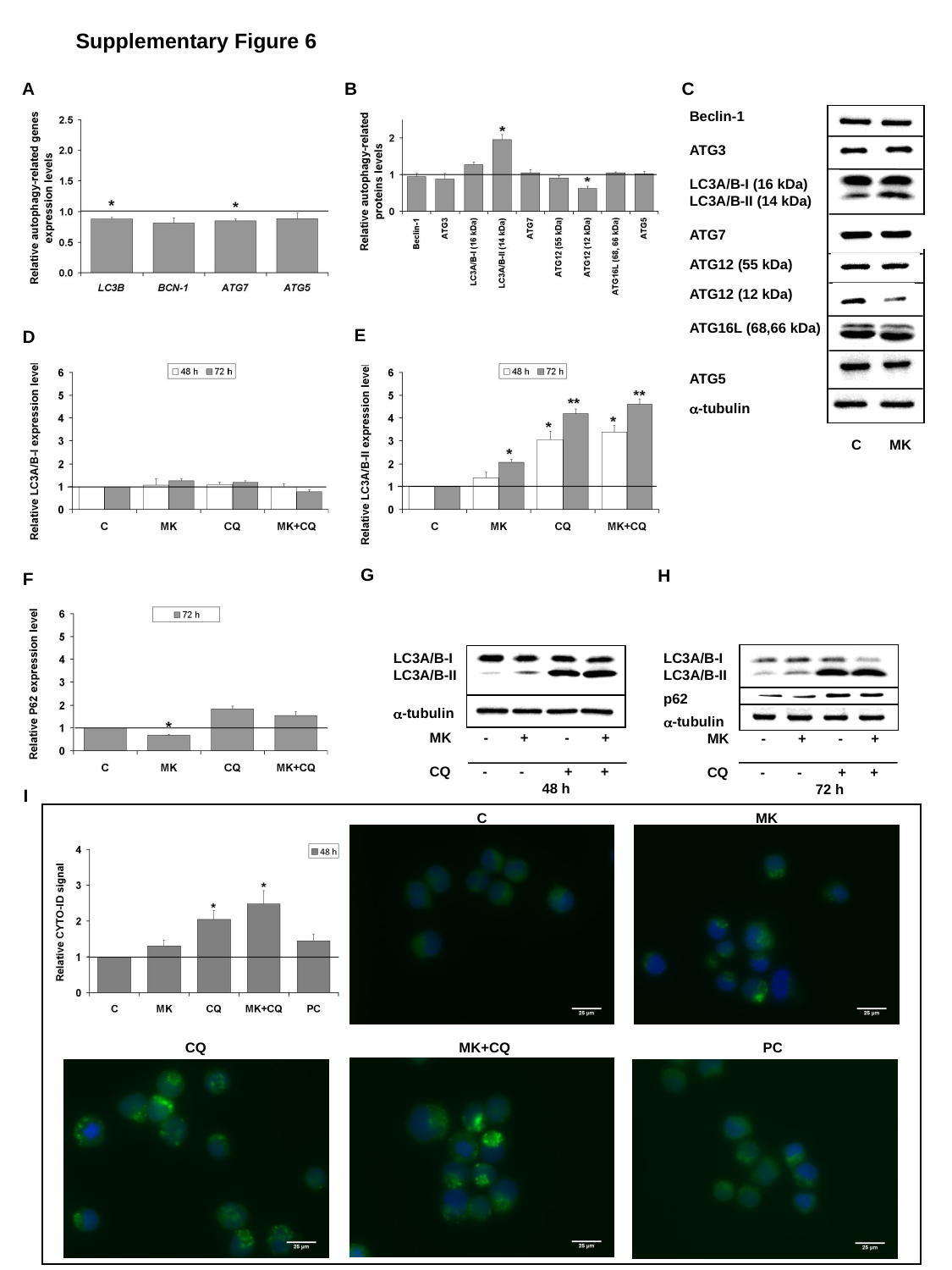

Supplementary Figure 6
A
B
C
Beclin-1
ATG3
LC3A/B-I (16 kDa)
LC3A/B-II (14 kDa)
ATG7
ATG12 (55 kDa)
ATG12 (12 kDa)
ATG16L (68,66 kDa)
ATG5
α-tubulin
 C MK
E
D
G
H
F
LC3A/B-I
LC3A/B-II
α-tubulin
LC3A/B-I
LC3A/B-II
p62
α-tubulin
 MK - + - +
 CQ - - + +
 48 h
 MK - + - +
 CQ - - + +
 72 h
I
 C MK
CQ MK+CQ PC
