# Supplementary material for: GD2 ganglioside-binding antibody 14G2a and specific aurora A kinase inhibitor MK-5108 induce autophagy in IMR-32 neuroblastoma cells
Source: Apoptosis. 2018 Jul 19;23(9):492–511. doi: 10.1007/s10495-018-1472-9 (PMC6153511; doi:10.1007/s10495-018-1472-9)
Supplement: Supplementary file 2 — Supplementary material 2 (DOC 47 KB) [file 10495_2018_1472_MOESM2_ESM.doc]

**Supp. Fig. 1. Autophagy in the 14G2a mAb-treated CHP-134 neuroblastoma cells.** (A) Relative gene expression level of *LC3B,* *BCN-1*, *ATG12* and *ATG5* in the 14G2a mAb-treated CHP-134 cells (in concentration of 40 μg/ml) as compared to the control (PBS-treated cells) assessed at 48 h by RT-qPCR. *RPS13* cDNA was used as the reference. (B) Relative protein expression of Beclin-1, ATG3, LC3A/B-I, LC3A/B-II, ATG7, ATG12, ATG16L was measured in CHP-134 cells at 48 h by western blot and normalized to α-tubulin. Expression of proteins and their respective genes in the control cells equals 1 (black baseline). (C) Representative immunoblots are presented. (D) Relative LC3A/B-I expression affected by the 14G2a or/and CQ treatment in CHP-134 cells as assessed by western blot. Cells were pre-treated with 10 μM CQ for 1.5 h and subsequently treated with the 14G2a mAb or PBS (control) for 48 and 72 h. (E) LC3A/B-II – estimated autophagy flux affected by the 14G2a mAb or/and CQ treatment in CHP-134 cells as assessed by western blot. (F) Relative P62 expression affected by the 14G2a mAb or/and CQ treatment in CHP-134 cells as assessed by western blot. (G, H) Representative immunoblots are presented. C – control, PBS-treated cells, 14G2a – the 14G2a mAb-treated cells, CQ – chloroquine-treated cells. P-values for t test were as follow: p<0.05 (*).

**Supp. Fig. 2.** **Relevance of autophagy flux in the 14G2a-treated CHP-134 cells.** Cells were pre-treated with 10 μM CQ for 1.5 h and subsequently treated with the 14G2a mAb or PBS (control). (A) CYTO-ID – estimated autophagy flux CHP-134 cells. Relative mean fluorescence intensity of the CYTO-ID dye was measured at 24, 48 and 72 h by a microplate reader and divided by ATP level of the respective groups of cells. (B) CHP-134 cells were stained with CYTO-ID and Hoechst 33342, fixed and visualized under the fluorescence microscope. Relative mean fluorescence intensity of CYTO-ID-stained autophagic compartments was quantified in five randomly selected photomicrographs (taken using a 40x objective). (C) Localization of CYTO-ID and Hoechst 33342 fluorescence dyes in CHP-134 cells was assessed using fluorescence microscope at 48 h. Scale bar – 25 μm. C – control, PBS-treated cells; 14G2a – the 14G2a mAb-treated cells; CQ – chloroquine-treated cells; PC – positive control, cells grown in amino acids-free medium for 24 h. P-values for t test were as follow: p<0.05 (*).

**Supp. Fig. 3.** **Assessment of autophagy features of the 14G2a-treated CHP-134 cells by transmission electron microscopy.** CHP-134 cells were pre-treated with 10 μM CQ for 1.5 h and subsequently treated with the 14G2a mAb or PBS for 48 h, and visualized by transmission electron microscope. The representative images account for exemplary photos of cells selected from at least 20 other photos of cells visualized under the microscope for each type of treatment. Autophagosomes (arrows) and residual bodies (arrowheads) are scattered along a cytoplasm. Scale bar – 2 μm. N – nucleus, M – mitochondria. C – control, PBS-treated cells; 14G2a – the 14G2a mAb-treated cells; CQ – chloroquine-treated cells.

**Supp. Fig. 4. Effects of combinatorial treatment of neuroblastoma cells with the 14G2a mAb and autophagy inhibitors.** IMR-32 (A), CHP-134 (C), LA-N-1 (E) and LA-N-5 (G) cells were treated for 1.5 h with indicated concentrations of CQ or/and in combination with the 14G2a mAb (40 μg/ml)/PBS for 72 h. IMR-32 (B), CHP-134 (D), LA-N-1 (F) and LA-N-5 (H) cells were treated for 1.5 h with the indicated concentrations of Baf or/and in combination with the 14G2a mAb (40 μg/ml)/PBS for 72 h. Cell cytotoxicity was determined by measuring ATP content, and compared to respective controls treated with appropriate diluents (water for CQ or DMSO for Baf). Standard errors of the mean bars were omitted from the graph for the 14G2a mAb-treated cells for clarity, but were less <0.2 for all data points. P-values for t test were as follow: p<0.05 (*), p<0.01 (**) and refer to the 14G2a mAb and inhibitors-combined treatment, as compared to inhibitors alone.

**Supp. Fig. 5. Effects of stable silencing of the *PHLDA1* gene on expression of autophagy and apoptosis-associated proteins in selected CHP-134 clones.** (A) The expression of autophagy-associated proteins (LC3A/B-I, LC3A/B-II, P62, ATG3, Beclin-1, and ATG7) was measured in *PHLDA1*-silenced (S5, S6, S16, and S17), Mock1-2, and WT cells at 48 h by western blot and normalized to α-tubulin. (B) The expression of apoptosis-associated proteins (PARP, cleaved PARP, and cleaved caspase 3) was measured in *PHLDA1*-silenced (S5, S6, S16, and S17), Mock1-2 and WT cells at 48 h by western blot.

**Supp. Fig. 6. Autophagy in MK-5108-treated CHP-134 cells.** (A) Relative gene expression level of *LC3B,* *BCN-1*, *ATG7*, and *ATG5* in MK-5108-treated CHP-134 cells (0.1 μM), as compared to the control (DMSO-treated cells), assessed at 72 h by RT-qPCR. *RPS13* cDNA was used as the reference. (B) Relative protein expression of Beclin-1, ATG3, LC3A/B-I, LC3A/B-II, ATG7, ATG12, ATG16L, and ATG5 was measured by western blot and normalized to α-tubulin. (C) Representative immunoblots are presented. (D) Relative LC3A/B-I expression affected by MK-5108 or/and CQ treatment assessed by western blot. CHP-134 cells were pre-treated with 10 μM CQ for 1.5 h and subsequently treated with 0.1 μM MK-5108 or DMSO (control, inhibitor solvent) for 48 and 72 h. (E) LC3A/B-II – estimated autophagy flux affected by MK-5108 or/and CQ treatment assessed by western blot. (F) Relative P62 expression affected by MK-5108 or/and CQ treatment as assessed by western blot. (G, H) Representative immunoblots are presented. (I) CHP-134 cells were stained with CYTO-ID and Hoechst 33342, fixed and visualized under the fluorescence microscope at 48 h. Relative mean fluorescence intensity of CYTO-ID-stained autophagic compartments was quantified in five randomly selected photomicrographs (taken using a 40x objective). Scale bar – 25 μm. C – control; DMSO-treated cells; MK – MK-5108-treated cells; CQ – chloroquine-treated cells, PC – positive control, cells grown in amino acids-free medium for 24 h. P-values for t test were as follow: p<0.05 (*), p<0.01 (**).
